# Alternative Devices for Heart Rate Variability Measures: A Comparative Test–Retest Reliability Study

**DOI:** 10.3390/bs11050068

**Published:** 2021-05-02

**Authors:** Jacquelin M. Killian, Rachel M. Radin, Cubby L. Gardner, Lalon Kasuske, Kylee Bashirelahi, Dominic Nathan, David O. Keyser, Christopher J. Cellucci, David Darmon, Paul E. Rapp

**Affiliations:** 1 711th Human Performance Wing, Air Force Research Laboratory, Wright Patterson Air Force Base, WPAFB, OH 45433 USA; Jacqueline.Killian@us.af.mil; 2 Department of Psychiatry and Behavioral Medicine, Uniuversity of California, San Francisco, San Francisco, CA 94143, USA; rachel.radin@ucsf.edu; 3 Defense Health Headquarters, Falls Church, VA 22042 USA; cubby.l.gardner.mil@mail.mil; 4 Center for Nursing Science and Clinical Inquiry, Walter Reed National Military Medical Center, Bethesda, MD 20814, USA; lalon.m.kasuske.mil@mail.mil; 5 Traumatic Injury Research Program, Uniformed Services University, Bethesda, MD 20814, USA; kyleebadii@gmail.com (K.B.); david.keyser@usuhs.edu (D.O.K.); 6 Center for Neuroscience and Regenerative Medicine, Uniformed Services University and the Henry M Jackson Foundation for the Advancement of Military Medicine, Bethesda, MD 20814, USA; dominic.nathan.ctr@usuhs.edu; 7 Aquinas LLC, Berwyn, PA 19312 USA; cellucci@gmail.com; 8 Department of Mathematics, Monmouth University, West Long Branch, NJ 07764, USA; ddarmon@monmouth.edu

**Keywords:** heart rate variability, portable heart rate monitor, HRV, test–retest reliability, intraclass correlation coefficients, photoplethysmography

## Abstract

Using healthy adult participants, seven measures of heart rate variability were obtained simultaneously from four devices in five behavioral conditions. Two devices were ECG-based and two utilized photoplethysmography. The 140 numerical values (measure, condition, device) are presented. The comparative operational reliability of the four devices was assessed, and it was found that the two ECG-base devices were more reliable than the photoplethysmographic devices. The interchangeability of devices was assessed by determining the between-device Limits of Agreement. Intraclass correlation coefficients were determined and used to calculate the standard error of measurement and the Minimal Detectable Difference. The Minimal Detectable Difference, MDD, quantifies the smallest statistically significant change in a measure and is therefore critical when HRV measures are used longitudinally to assess treatment response or disease progression.

## 1. Introduction

It has been suggested that it may be possible to use measures of heart rate variability (HRV) as quantitative physiological biomarkers in the longitudinal assessment of treatment. Several devices are available for HRV studies, and several different measures of HRV can be calculated from the electrocardiogram. The values of these measures will be different in different behavioral conditions. If HRV is to be used to assess longitudinal change, it is essential to know if an observed change in a measure is statistically significant: is the observed change greater than the Minimal Detectable Difference for that measure in that behavioral condition? Addressing this question requires test–retest data. This study compared seven measures calculated in five behavioral conditions from ECGs recorded simultaneously with four different devices. As ECG records from the four devices were obtained simultaneously, direct comparison of the resulting measures of heart rate variability was possible. The following questions are addressed:

1. What is the comparative operational reliability of the four devices?
2. What are the numerical values of the seven HRV measures in the five behavioral conditions as determined by each device?
3. Can different devices be used interchangeably? To address this question, it was necessary to determine inter-device agreement by determining the Bland–Altman Limits of Agreement (LOA). This determination is clinically important because it advises the clinician of the possible implications of replacing one device with another in the course of treatment.
4. What constitutes a significant change in an HRV measure? Addressing this question required a determination of test–retest reliability across repeated measurements for the seven measures in five conditions for each device. Longitudinal clinical use requires a quantitative assessment of test–retest reliability as quantified by the intraclass correlation coefficient. Determination of the intraclass correlation coefficient requires multiple measurements from a clinically stable population. The requirement to use a clinically stable population motivates the use of a healthy control population in these studies. Intraclass correlation coefficients were then used to calculate the Standard Error of Measurement and the Minimal Detectable Difference (MDD). The Minimal Detectable Difference is the smallest change that could be identified as statistically significant and is therefore critical when HRV measures are used longitudinally to assess treatment response or disease progression. The prior literature assessing HRV test–retest reliability [1,2,3] presents encouraging retest reliability, but these studies do not include the calculation of the clinically important Minimal Detectable Difference. We note, however, that Williams et al. [4] report the related Standard Error of Measurement that can be used to calculate the Minimal Detectable Difference.

Broadly stated, two classes of device are used in HRV research. Both the EPA6 and the BioPatch device used here obtain heart rate from transcutaneous bioelectrical impulses. In contrast, the HeartMath devices use pulse oximetry technology or photophlethysmography (PPG). Heart rate detected by PPG relies on the pulse wave propagated through the vascular tree following ventricular contraction; thus, measuring the pulse wave as a surrogate for the ECG. Variability derived from PPG signal is often referred to, more correctly, as pulse rate variability (PRV). While there are several studies that comparison test different technologies for measuring heart rate, the literature comparing heart rate variability assessments is smaller. When comparing ECG-based devices and photoplethysmography two distinct, albeit related, questions should be addressed: the agreement of acquired HRV measures and the comparative longitudinal test–retest reliability of these measures. In response to the first question, the prior literature offers contrasting results. Lu and Yang [5] found that in laboratory conditions ECG and PPG give highly correlated HRV measures, but in more naturalistic conditions the PPG technology was vulnerable to motion artifacts. Guzik et al. [6] found statistically significant differences in HRV measures calculated form ECG records and mobile device data. In contrast, Vovkanych et al. [7] found good agreement between ECGs as did Huang et al. [3] in a study that included a self-selected walking velocity task. Correia et al. [8] compared ECG and PPG-derived HRV measures and found that agreement varied depending on the HRV measures. A detailed specification of these measures can be found in their paper. Interestingly, they found good agreement in low frequency HRV spectral measures and poor agreement in high frequency measures. The most systematic comparison of ECG and portable device HRV measures is the meta-analysis of twenty three studies in Dobbs et al. [9]. They found that HRV measures from portable devices differed from the ECG. The overall effect was small but varied greatly from study to study. This leaves unaddressed the question of the comparative test–retest reliability of ECG and PPG-based measures of heart rate variability. Between session differences in, for example, ear clip of chest strap placement and the sensitivity to motion identified by Lu and Yang [5] suggest that PPG measures could present lower test–retest reliability. This question is addressed in this study.

## 2. Materials and Methods 

### 2.1. Sample

Fourteen healthy participants (mean age 38.6 ± 7.2 y, range: 20–48 y, 8 male, 6 female) volunteered for the study. All participants were affiliated with the graduate programs at the Uniformed Services University as either enrolled students or undergraduate student volunteers. Medication use was recorded for each candidate participant. Individuals taking beta-blockers, benzodiazepines or antipsychotic medications were not included in the study. Exclusion criteria included cardiac history (heart arrhythmia, internal or external defibrillator use) and medications that could impact cardiac activity. The Paced Auditory Serial Addition Task was administered as part of the study. Compared against age-appropriate normative values (Wiens et al., 1997) all participants obtained normal or high normal scores. Prior to testing, participants gave written informed consent to participate in this Institutional Review Board approved study. Participants were not paid or compensated for their participation. All study procedures were conducted in accordance with human participant protections regulations required by ethical laws and regulations set forth by the Declaration of Helsinki and the Common Rule. All participants scheduled their first and second session as their academic or work schedule would allow and were instructed to report to the Traumatic Injury Research Program (TIRP) laboratory to accomplish the estimated one-hour protocol on each session day.

### 2.2. Procedure

Participants were required to attend two sessions in the laboratory separated by at least 24 h and no more than twelve days. At the start of the session, each participant was instrumented to allow for the simultaneous recording of ECG via 4 pathways. Following instrumentation, as detailed below, the participant was instructed to relax alone for 5 min prior to the collection of data to allow for autonomic nervous system stabilization that may have been perturbed with the instrumentation procedure. The first protocol segment was a seated rest segment in which participants were instructed to sit still with eyes closed (without falling asleep) for ten minutes. In the second segment participants took the Paced Auditory Serial Addition Task [10], an 8 min cognitive stressor. The third time segment consisted of a second ten-minute rest period with eyes closed, followed by the fourth time segment of paced breathing for five minutes. Paced breathing was achieved by breathing slowly in and out using a consistent and slow count to five, with the pace determined by the participant. Finally, during the fifth time segment participants were asked to stand (from seated position) with arms comfortably at their sides and eyes closed as an orthostatic challenge for five minutes. The duration of the ECG records was the same as the duration of the task. All recordings took place in the electromagnetically shielded laboratory to control for extraneous electrical noise.

### 2.3. Signal Acquisition

EPA6: A standard 3 lead ECG configuration was achieved using the Sensorium ElectroPhysiology Amplifier system (EPA6, Sensorium, Inc. Charlotte, VT, USA), a general-purpose amplifier, set to sample at a rate of 2 KHz. Participants were monitored using three channels (under mid right clavicle, left ankle, and left wrist as the ground electrode). ECG recordings were obtained for each specific segment of the protocol. The EPA6, a high-resolution data acquisition system, was used to set the standard with which to compare the other recording modes, as described below. 

emWave 2: The emWave 2 device (HeartMath LLC, Boulder Creek, CA, USA) captures heart rate using photophlethysmography (PPG) using a probe that is clipped to the participant’s earlobe and records at 200 Hz. Two emWave devices were attached to the participant (separate earlobes); one was used as a handheld device (HM_HH_) while the other emWave 2 device was attached to a laptop PC (HM_PC_). Both emWave 2 devices recorded continuously from start to end of protocol. Each of the five protocol segments were identified and separated off-line for analysis.

BioPatch: The third device used was the BioPatch (Zephyr Technology Inc., Zephyr Technology Inc., Baltimore, MD, USA), which acquires transcutaneous bioelectrical impulses to measure 3 lead ECG signal and records at 250 Hz. The BioPatch attaches to the participant’s chest with 2 standard disposable ECG pads and is placed over the sternum at the fourth intercostal space. The BioPatch recorded continuously from start to end of protocol. Each of the five protocol segments were identified and separated off-line for analysis.

### 2.4. Signal Analysis

The digitized ECG data from each of the four modes of HRV data collection were inputted into a custom-written MATLAB program (MathWorks, Inc., Natick, MA, USA) for identification and extraction of the inter-beat-interval (IBI) series, by determining the difference between successive peaks of the QRS complexes. The IBI data were then pre-processed using the Kubios Software package, Version 2.0 [11]. Pre-processing consisted of correcting the trends in the IBI time series using smooth priors and identifying non-physiological outliers to be removed with medium level of correction. In addition, the corrected IBI time series was re-sampled at 4 Hz. Subsequently, HRV time and frequency analysis of the inter beat interval data sequence was calculated using the Kubios software package. For analysis purposes, the time-domain variables of interest selected for comparisons were the mean of the R-to-R intervals (Mean RR), the standard deviation of the R-to-R intervals (SDNN), mean heart rate (Mean HR); standard deviation of instantaneous heart values (STD HR); and the root mean square of successive differences (RMSSD). The accuracy of Kubios output was confirmed by independent calculations performed with our own software. The first frequency-domain variable of interest selected for comparisons was the LF/HF ratio, which is the ratio between Low Frequency and High Frequency power bands. The low frequency band was defined as 0.04 Hz to 0.15 Hz and the high frequency band was defined as 0.15 Hz to 0.4 Hz. While the LF/HF ratio is commonly reported in the clinical HRV literature, it should be noted that it is an imperfect measure of sympatho-vagal balance [12]. A second frequency domain measure was therefore incorporated into the study, the percent of power spectral density in the high frequency domain.

### 2.5. Limits of Agreement

Agreement comparing the EPA6 ECG, and the three portable devices was investigated using the first visit data. As Luiz and Szklo [13] note more than one statistical strategy can be used to assess the agreement of quantitative measurements. We utilized here the most commonly used procedure as introduced by Bland and Altman [14] for the case where an absence of a clinically significant systematic bias was assumed. Limits of agreement and the 95% confidence intervals for both the upper and lower limits of agreement were calculated. Provided that the differences determined by these bands are not clinically significant, two devices can be used interchangeably. The degree to which this difference is or is not clinically significant is a matter of clinical judgement. 

### 2.6. Intraclass Correlation Coefficients

Visit 1 to Visit 2 test–retest reliability was quantified by the intraclass correlation coefficient. Several definitions of the ICC have been proposed. Shrout and Fleiss [15] provided six definitions and McGraw and Wong [16] published ten definitions. Guidance for selection is given in Müller and Büttner [17] and in Koo and Li [18]. In this investigation we used ICC(2,1) [15] with the confidence intervals derived in Fleiss and Shrout [19]. The Supplement presents ICC(2,1) values with confidence intervals for seven measures, five behavioral conditions and four devices.

### 2.7. Minimal Detectable Difference

The intraclass correlation coefficients were used to calculate the longitudinal Standard Error of Measurement [20]. Numerical values are given in Table S5 of the Supplement. The Standard Error of Measurement was then used to calculate the Minimal Detectable Difference [20]. The Minimal Detectable Difference is of clinical interest. As noted in the Introduction, alterations in measures of heart rate variability associated with neuropsychiatric disorders are nonspecific and therefore of limited value diagnostically when used in isolation from other measures. A greater value may be realized in longitudinal application. Longitudinal use to assess change in clinical state requires quantification of a measure’s variability. The Minimal Detectable Difference is the smallest statistically important change and is therefore critical in longitudinal clinical assessments.

### 2.8. Statistical Analysis

Statistical analysis was performed with IBM SPSS 22.0 [IBM Corp., Armonk, NY, USA]. Procedures advocated by Behrens [21] were used to examine study variables to determine whether the assumptions of univariate and multivariate analyses were met. All data were screened for problems of outliers, skew, and kurtosis. Outliers (<5% of all data points) were Winsorized to fall 1.5 times the interquartile range below or above the 25th or 75th percentile. This strategy was used because it minimizes the influence of outliers on the characteristics of the distribution, minimally changes the distribution overall, and avoids potential bias associated with the elimination of outliers altogether. This correction did not significantly alter the direction or magnitude of any result.

To assess the reliability of the two HRV devices (HM and BioPatch) in comparison to the standard three-lead ECG (EPA6) first, the overall signal acquisition reliability of each device was examined based on the amount of data lost; second, we performed a comparison of data across the first and second session by estimating intraclass correlation coefficients; third we investigated inter-device agreement by estimating the limits of agreement and their corresponding confidence intervals; fourth we compared devices throughout each phase of each session; and lastly, we estimated a two-way intra-class correlation between the ECG device (EPA6) and each of the other devices.

For our primary analyses, reliability was assessed statistically using a series of linear mixed models with three fixed factors (device, segment, session) and a random subject effect to calculate the difference, if any, between devices and between sessions. Linear mixed models were run to compare time-domain and frequency-domain measures of HRV, as obtained from Kubios Version 2.0 [11]: Mean RR (ms), SDNN (ms), Mean HR (1/min), STD HR (1/min), RMSSD (ms), LF/HF ratio power (ms^2^) and high frequency power spectral density.

There were 4 devices compared: EPA6, BioPatch, HM_PC_, and HM_HH_. There were 5 task segments compared: initial rest session, stressor (PASAT), second rest session, paced breathing, and standing session. Finally, there were two sessions compared: session A and session B. Post hoc analyses were conducted as warranted, to ascertain differences between devices at each segment of a session, using univariate *F*-tests. General agreement between devices is described using the Bland–Altman method [14] where, the average of two methods is plotted along the horizontal axis and the difference of each device from this average is plotted along the vertical axis. Additionally, limits of agreement and the 95% confidence intervals of both the upper and lower limit of agreement were calculated using first visit data following the analysis in Bland and Altman, as was deemed appropriate for our primary analyses in order to compare how each device performed in comparison with our comparison measure (EPA6). Differences between devices, segments, and sessions were considered significant when *p*-values were <0.008333. Given that multiple comparisons were made, a Bonferroni adjusted alpha level of 0.01 (0.05/6) was used. All tests were for two-sided alternative hypotheses.

## 3. Results

### 3.1. Operational Reliability

Participants had an average of four days (range: 1–12 days) between session A and session B. Across all participants (n = 14), for session A and session B (140 session segments recorded in total across all participants), both the EPA6 and BioPatch devices evidenced 0% data loss (both devices recorded 140 out of 140 possible session segments in Kubios). By contrast, the HM_PC_ device exhibited data collection failures approximately 19% of the time (113 out of 140 sessions recorded) and the HM_HH_ device exhibited collection failures approximately 11% of the time (125 out of 140 sessions recorded). A detailed report of comparative operational reliability statistics is given in Table S1 of the Supplement.

### 3.2. Numerical Values of HRV Measures

**Figure 1:**
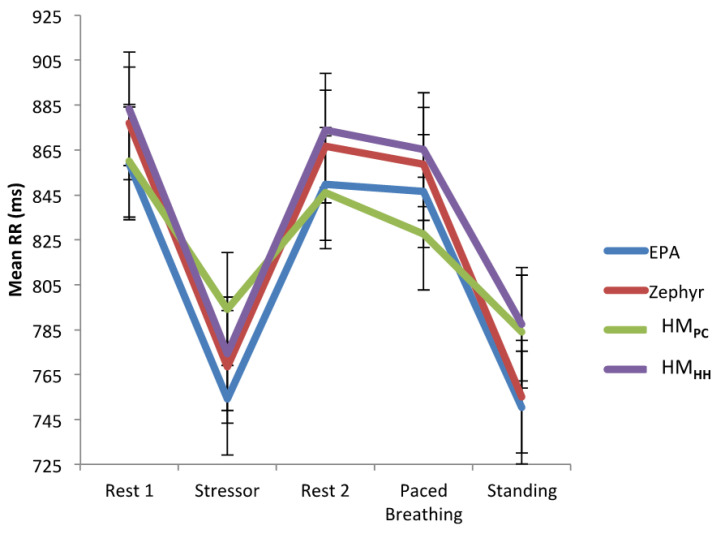
**Mean RR values:** When aggregating data across both sessions, there was a main effect of segment, indicating a general decrease in Mean RR from initial rest, in response to the stressor, followed by a general increase during the recovery portion of the paradigm and then a slight decrease in Mean RR during the standing segment (*F* = 65.42, *p* < 0.001); There appeared to be a main effect of device, such that HM_HH_ tended to display greater Mean RR values compared to EPA6 (*F* = 3.42, *p* = 0.018); and when examining the interaction between device and segment all 4 devices tended to vary in a similar pattern throughout the session (*F* = 0.75, *p* = 0.70). Data from a linear mixed model with two fixed factors (device, segment) and a random subject effect to calculate the difference between devices and between sessions. Analogous diagrams for five other HRV measures are in the Supplement.

Numerical values of the measures are report in Table S2 of the Supplement. The mathematical definitions of the time domain measures are given in the Appendix (Supplement). Using four devices, seven measures are reported (mean interbeat interval, the standard deviation of RR intervals, mean heart rate, the standard deviation of the heart rate, the root mean square of successive differences, the ratio of low frequency to high frequency spectral power of the RR interval spectrum, percent power spectral density in the high frequency band of the HRV spectrum). Results from five behavior conditions (Rest 1, Stressor, Rest 2, Paced Breathing and Standing) are reported in that table. Figure 1 shows the result for the mean RR interval across behavioral states. The corresponding diagrams for the other five measures are given in the Supplement.

### 3.3. Inter-Device Agreement: Limits of Agreement (LOA) of Portable Devices with Standard ECG System

**Figure 2:**
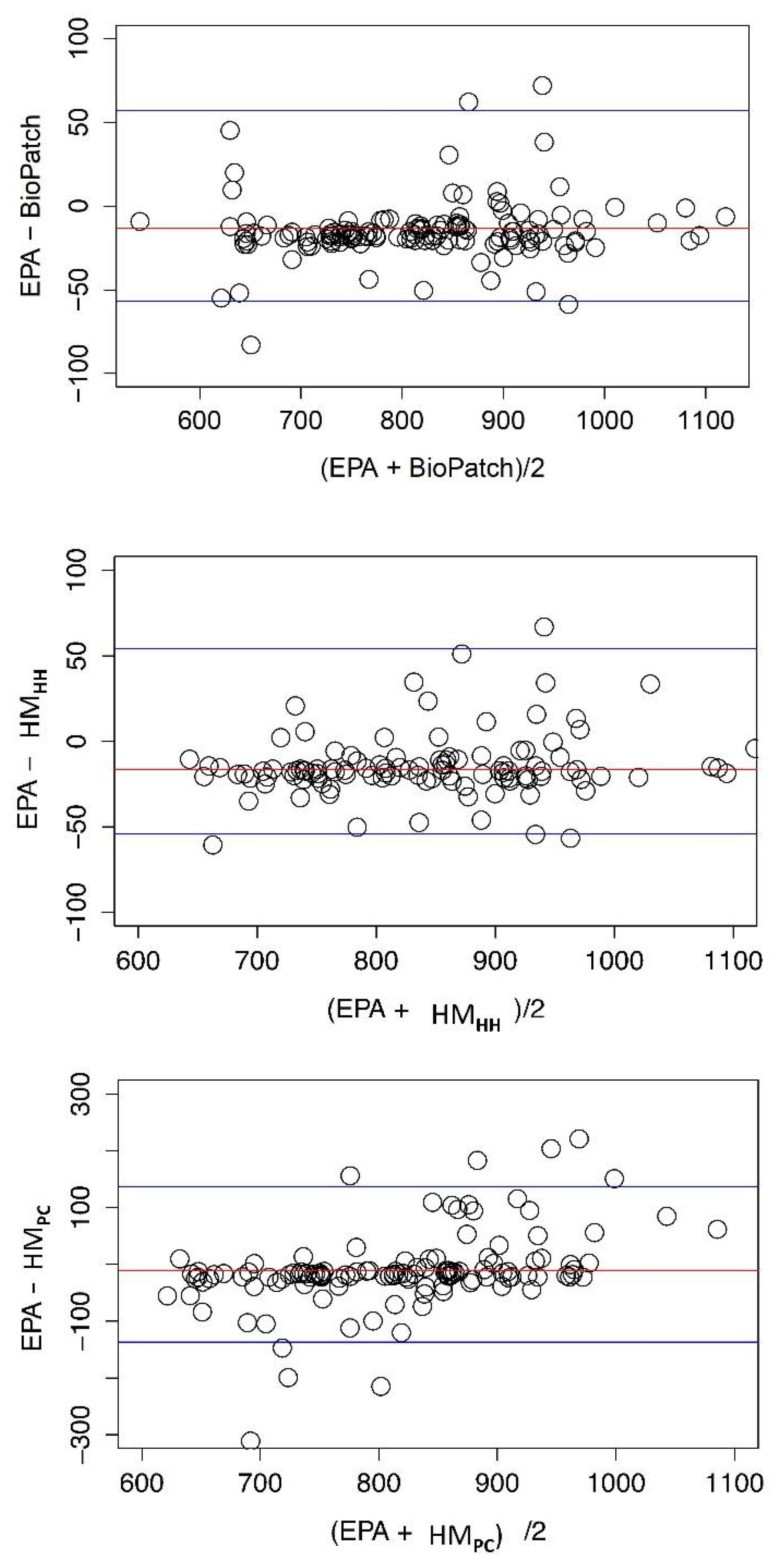
Inter-device agreement EPA6 and three portable devices. Agreement between the EPA6 and three portable devices using the Bland–Altman method is depicted (Bland and Altman, 1986). For any given measure and condition the average value obtained in the two methods is plotted on the x-axis and the difference is plotted on the y-axis. Data from all five behavioral conditions are shown in the diagram. Top: Comparison of the EPA6 and the BioPatch. Middle: Comparison of the EPA6 and the HeartMath handheld device. Bottom: Comparison of the EPA6 and the HeartMath device connect to a PC. Note that the specification of the y axis in this panel differs from that in the two preceding panels.

A detailed specification of the limits of agreement and their corresponding confidence intervals is given in the Supplementary Material. A summary is given in Table 1 which shows the Bland–Altman limits for seven measures averaged over five behavioral conditions. A detailed presentation is given in Table S6 of the Supplement. As outlined in Bland and Altman [14] a true 95% confidence band for inter-device agreement would be from the lower 95% confidence bound of the lower limit of agreement to the upper 95% confidence bound of the upper limit of agreement. Reference to the Supplementary Material indicates that the true 95% confidence interval can be significantly greater than the interval suggested by the upper and lower limits of agreement. It is seen that the band specified by the limits of agreement for the HeartMath device connected to a computer (EPA6-HM_PC_ in Table) is significantly greater than in the other comparisons. This is reflected in the graphical presentation in Figure 2. When interpreting limits of agreement and comparing them with the results of test–retest reliability (next section), it should be recalled that limits of agreement and measures of reliability measure are fundamentally different things. It is possible for devices to be highly reliable (self-consistent) but disagree with other devices giving very broad limits of agreement.

### 3.4. Test–Retest Reliability: Intraclass Correlation Coefficients, Standard Error of Measurement, Minimal Detectable Difference

There were no statistically significant differences between session A and session B when comparing Mean RR (*F* = 1.22, *df* = 1,11.5, *p* = 0.29), SDNN (*F* = 0.81, *df* = 1,11.8, *p* = 0.39), Mean HR (*F* = 1.31, *df* = 1,11.5, *p* = 0.28), SDNN (*F* = 0.08, *df* = 1,11.6, *p* = 0.79), RMSSD (*F* = 1.67, *df* = 1,11.4, *p* = 0.22), and LF/HF ratio power (*F* = 0.41, *df* = 1,11.1, *p* = 0.54) across all devices and segments. When aggregating data across both sessions, as expected, there was a main effect of segment, indicating a general decrease in all 6 HRV measures from initial rest, in response to the stressor, followed by a general increase during the recovery portion of the paradigm and then a slight decrease in HRV during the standing segment (Mean RR, *F* = 65.42, *p* < 0.001; SDNN, *F* = 26.02, *p* < 0.001; Mean HR, *F* = 62.04, *p* < 0.001; STD HR, *F* = 19.62, *p* < 0.001; RMSSD, *F* = 2.97, *p* = 0.02; and LF/HF ratio power, *F* = 17.64, *p* < 0.001). These findings confirm a physiological stress response to our laboratory paradigm (the PASAT).

There appeared to be a main effect of device, such that HM_PC_ tended to display greater HRV values compared to EPA6 and BioPatch across several HRV measurements (SDNN, *F* = 9.32, *p* < 0.001; STD HR, *F* = 8.48, *p* < 0.001; RMSSD, *F* = 41.76, *p* < 0.001; and decreased LF/HF ratio power, *F* = 10.65, *p* < 0.001). In particular, post hoc analyses indicated HM_PC_ tended to be higher than EPA6 during the standing segment. (*p* < 0.001). Additionally, HM_HH_ tended to display greater HRV compared to EPA6 and BioPatch across one HRV measurement (RMSSD, *F* = 41.76, *p* < 0.001..

However, when examining the interaction between device and segment (our primary outcome of interest) all devices tended to vary in a similar pattern throughout the session (Mean RR, *F* = 1.24, *p* = 0.26; SDNN, *F* = 0.75, *p* = 0.70; Mean HR, *F* = 1.32, *p* = 0.20; STD HR, *F* = 0.53, *p* = 0.90; RMSSD, *F* = 1.00, *p* = 0.45; and LF/HF ratio power, *F* = 1.01, *p* = 0.44). Figure 2 shows the graphical depictions of the main effects of device and segment, as well as the interaction between device x segment for each HRV measurement.

The Minimal Detectable Difference is the smallest statistically important change and is therefore critical in longitudinal clinical assessments. Table 2 gives a summary of minimal detectable differences averaged across behavioral conditions. A detailed presentation is given in Table S3 of the Supplement.

## 4. Discussion

The objectives of this study were enumerated in the introduction. The corresponding results are as follows:

1. Comparative Operational Reliability.

As detailed in Table S1 of the Supplement, the EPA6 and BioPatch devices were found to be highly reliable. The probability of failure (a lost record) with the PC-connected HeartMath device was 19%, and the probability of a lost record with the hand-held HeartMath devices was 11%.

2. Numerical values of HRV measures.

The motivating question was, what are the numerical values of the seven HRV measures in the five behavioral conditions as determined by each device? The one hundred forty values with standard deviations are presented in Table S2 of the Supplement.

3. Can devices be used interchangeably?

This question was addressed by determining the inter-device limits of agreement. A summary is given in Table 1 and a detailed specification is given in Table S6 of the Supplement. The determination of the Minimal Detectable Difference and a determination of the Limits of Agreement for each condition (device, measure, behavior) increases the utility of the devices when used individually. The Limits of Agreement advises a clinician of the possible implications of replacing one device with another. The acceptability of a replacement is a clinical judgment.

4. What constitutes a significant change in an HRV measure?

Addressing this question required determination of the Minimal Detectable difference. A summary (mean ± standard deviation) is given in Table 2. The detailed report is in Table S3 of the Supplement. As previously noted, the Minimal Detectable Difference advises a clinician if an observed change in a measure is statistically significant.

The measurement reliability results of this investigation are concordant with published literature comparing methods of assessing HRV [4,22,23], and suggest that the assessed devices are equally capable of collecting valid measures in normal healthy subjects. All devices tended to vary in a similar pattern across segments, compared to the standard method (EPA6). Similarly, all devices showed good test retest reliability when comparing Session A to Session B. The concordance of intra-class correlation (ICC) suggests that these devices demonstrate acceptable and similar validity when used to collect heart rate signal that is filtered and further analyzed with Kubios HRV analysis software. Our findings lead us to consider both the emWave 2 and the Biopatch as valid and reliable for use in both research and clinical settings with the caution that the HeartMath devices are sensitive to motion artifact, for this reason throughout our protocol, participants were reminded to remain still while seated and while standing to reduce incidence of motion artifact. For studies involving movement, the Biopatch would be a more suitable portable alternative for collection of ECG data.

Several limitations of this study should be noted.

1. The preliminary nature of this underpowered exploratory study is explicitly recognized. Zou [24] provided an estimate of the sample size required in a test–retest reliability study. He determined that in a study with two assessments for each participant and an approximate value of the intraclass correlation coefficient of 0.6, a 95% confidence interval with an assurance probability of 80% would require 183 participants. The wide confidence intervals seen with our estimates of the intraclass correlation coefficients reflect the small sample size of this study. Similarly, Liao [25] has investigated sample size for agreement studies and concluded that a sample size of at least 32 was required.

2. The Standard Error of Measurement and the Minimal Detectable Difference are distribution determined measures of statistical significance. That is, they are calculated from the numerical results of device measurements and are determined without reference to patient report. Specifically, the Minimal Detectable Difference is not the Minimal Clinically Important Difference. The determination of the Minimal Clinically Important Difference (MCID) requires an anchor-based method [20]. As summarized by Portney and Watkins [20], “In an anchor-based approach the magnitude of a change score is interpreted according to some clinical criterion or ‘anchor’ that is assumed to have an inherent meaning. A common anchor is the patient’s ordinal rating of improvement or decline.” A specific example of a commonly used clinical anchor is the Global Rating Scale, GRS. Using a GRS as a clinical anchor, the Minimal Clinically Important Difference can be determined in a five step process which is outlined in the Supplement.

3. Stratford et al. [26] report that the Standard Error of Measurement and hence the Minimally Detectable Difference can vary depending on the time interval between assessments. In this study, laboratory visits were separated by at least twenty-four hours and no more than twelve days. The results listed here will not necessarily generalize to longer duration longitudinal studies.

4. It should be remembered that reliability estimates are population specific. Changes that are significant in a healthy adult population (the population that participated in this study) are not necessarily significant in other populations. This is particularly true of neuropsychiatric patients. The behavior of an injured or diseased central nervous system can be highly variable. This has been known at least since Head’s pioneering work in behavioral neurology [27] and is vividly demonstrated by Bleiberg’s investigation of neuropsychological test–retest reliability following traumatic brain injury [28].

5. Only seven measures of heart rate variability are reported here. While these seven are the most commonly reported, a large number of additional measures were not investigated.

## Figures and Tables

**Table 1:** Bland–Altman limits of agreement for seven measures averaged over five behavioral conditions are shown. A detailed specification for each behavioral condition which includes the 95% confidence intervals of the lower and upper limits of agreement is given in the supplementary material.

	EPA6-BioPatch	EPA6-HM_PC_	EPA6-HM_HH_
Mean RR (ms)	[−46.04, 18.03]	[−133.63, 95.12]	[−71.14, 28.74]
SDNN (ms)	[−36.36, 33.02]	[−63.90, 44.73]	[−30.23, 33.99]
HR_Mean_ (1/min)	[−2.18, 4.77]	[−8.99, 14.22]	[−3.06, 7.10]
STD HR (1/min)	[−5.15, 4.33]	[−5.31, 3.43]	[−2.40, 2.69]
RMSSD (ms)	[−34.64, 25.25]	[−95.48, 32.73]	[−34.33, 25.04]
LF/HF Power	[−9.81, 13.47]	[−5.85, 14.02]	[−8.74, 13.04]
High frequency %PSD	[−40.00, 48.68]	[−67.57, 40.21]	[−35.12, 45.37]
Low frequency %PSD	[−18.42, 26.15]	[−19.55, 59.76]	[−18.91, 24.47]

**Table 2:** Summary: Minimal detectable differences averaged across behavioral conditions (Mean ± Standard Deviation).

	EPA6	BioPatch	HM_PC_	HM_HH_
Mean RR (ms)	140.11 ± 20.81	131.88 ± 19.40	137.20 ± 19.80	129.62 ± 9.26
SDNN (ms)	31.19 ± 15.57	34.33 ± 21.52	26.62 ± 6.55	23.87 ± 6.34
HR_Mean_ (1/min)	13.85 ± 3.24	12.69 ± 2.32	12.54 ± 1.96	12.55 ± 3.04
STD HR (1/min)	2.883 ± 1.383	3.173 ± 1.788	3.044 ± 0.582	2.452 ± 0.415
RMSSD (ms)	37.53 ± 8.91	44.70 ± 23.52	38.13 ± 6.65	33.50 ± 8.53
LF/HF Power	6.861 ± 4.594	6.631 ± 4.858	4.896 ± 2.507	8.365 ± 7.601
HF Power (%PSD)	25.774 ± 6.284	21.486 ± 1.716	33.826 ± 8.110	29.726 ± 8.390
LF Power (% PSD)	29.100 ± 7.315	24.106 ± 3.707	32.658 ± 7.915	31.680 ± 9.291

## Data Availability

Request for data should be directed to the first author. Availability is subject to Department of Defense policies concerning human research data.

## Supplementary Materials

The following are available online at https://www.mdpi.com/article/10.3390/bs11050068/s1. Appendix: Determination of the Minimal Clinically Importance Difference using a Global Rating Scale as the clinical anchor. Figure S1. Standard deviation of RR values for four devices in five behavioral conditions (SDNN); Figure S2. Root mean square of successive difference values for four devices in five behavioral conditions (RMSSD); Figure S3. Standard deviation of instantaneous heart values for four devices in five behavioral conditions (STD HR); Figure S4. Mean heart rate values for four devices in five behavioral conditions (Mean HR); Figure S5. Low frequency to high frequency ratio values for four devices in five behavioral conditions (LF/HF); Table S1. Operational reliability of the four devices; Table S2. Numerical values of HRV measures; Table S3. Minimal detectable differences with confidence intervals; Table S4. Intraclass correlation coefficients; Table S5. Standard error of measurement; Table S6. Bland–Altman limits of agreement.

## References

[1] Eikeseth F.F., Saetren S.S., Benjamin B.R., Eikenaes I.U.-M., Sutterlin S., Hummelen B. (2020). The test-retest reliability of heart rate functioning and its association with personality functioning. Front. Psychiatry.

[2] Hoffmann B., Flatt A.A., Silva LE V., Mlynczak M., Baranowski R., Dziedzic E., Werner B., Gasior J.S. (2020). A pilot study of the reliability and agreement of heart rate, respiratory rate, and short-term heart rate variability in elite modern pentathlon athletes. Diagnostics.

[3] Huang C.-J., Chan H.-L., Chang Y.-J., Chen S.-M., Hsu M.-J. (2021). Validity of the Polar V800 Monitor for Assessing Heart Rate Variability in Elderly Adults under Mental Stress and Dual Task Conditions. Int. J. Environ. Res. Public Health.

[4] Williams D.P., Jarczok M.N., Ellis R.J., Hillecke T.K., Thayer J.F., Koenig J. (2017). Two-week test-retest reliability of the Polar RX800CX to record hart rate variability. Clin. Physiol. Funct. Imaging.

[5] Lu G., Yang F. (2009). Limitations of oximetry to measure heart rate variability measures. Cardiovasc. Eng..

[6] Guzik P., Piekos C., Pierog O., Fenech N., Krauze T., Piskorski J., Wykretowicz A. (2018). Classic electrocardiogram-based and mobile technology derived approaches to heart rate variability are not equivalent. Int. J. Cardiol..

[7] Vovkanych L., Boretsky Y., Sokolovsky V., Berhtraum D., Krass S. (2020). Validity of the software-hardware complex “rhythm” for measuring the rr intervals and heart rate variability at rest. J. Phys. Educ. Sport.

[8] Correia B., Dias N., Costa P., Pêgo J.M. (2020). Validation of a Wireless Bluetooth Photoplethysmography Sensor Used on the Earlobe for Monitoring Heart Rate Variability Features during a Stress-Inducing Mental Task in Healthy Individuals. Sensors.

[9] Dobbs W.C., Fedewa M.V., MacDonald H.V., Holmes C.J., Cicone Z.S., Plews D.J., Esco M.R. (2019). The accuracy of acquiring heart rate variability from portable devices: A systematic review and meta-analysis. Sports Med..

[10] Gronwall D.M. (1977). Paced auditory serial-addition task: A measure of recovery from concussion. Percept. Motor Skills.

[11] Tarvainen M.P., Niskanen J.-P. Kubios HRV User’s Guide. http://kubios.uku.fi.

[12] Billman G.E. (2013). The LF/HF ratio does not accurately measure cardiac sympatho-vagal balance. Front. Physiol..

[13] Luiz R.R., Szklo M. (2005). More than one statistical strategy to assess agreement of quantitative measurements may be usefully reported. J. Clin. Epidemiol..

[14] Bland J.M., Altman D.G. (1986). Statistical methods for assessing agreement between two methods of clinical measurement. Lancet.

[15] Shrout P.E., Fleiss J.L. (1979). Intraclass correlations: Uses in assessing rater reliability. Psychol. Bull..

[16] McGraw K.O., Wong S.P. (1996). Forming inferences about some intraclass correlation coefficients. Psychol. Methods.

[17] Müller R., Büttner P. (1994). A critical discussion of intraclass correlation coefficients. Stat. Med..

[18] Koo T.K., Li M.Y. (2016). A guideline of selecting and reporting intraclass correlation coefficients for reliability research. J. Chiropr. Res..

[19] Fleiss J.L., Shrout P.E. (1978). Approximate interval estimation for a certain intraclass correlation coefficient. Psychometrika.

[20] Portney L.G., Watkins M.P. (2009). Foundations of Clinical Research. Applications to Practice.

[21] Behrens J.T. (1997). Principles and procedures of exploratory data analysis. Psychol. Methods.

[22] Jan H.-Y., Chen M.-F., Fu T.-C., Lin W.-C., Tsai C.-L., Lin K.-P. (2019). Evaluation of coherence between ECG and PPG derived parameters on heart rate variability and respiration in healthy volunteers with/without controlled breathing. J. Med Biol. Eng..

[23] Schafer A., Vagedes J. (2013). How accurate is pulse rate variability as an estimate of heart rate variability? A review on studies comparing photoplethysmographic technology with an electrocardiogram. Int. J. Cardiol..

[24] Zou G.Y. (2012). Sample size formulas for estimating intraclass correlation coefficients with precision and assurance. Stat. Med..

[25] Liao J.J. (2010). Sample size calculations for an agreement study. Pharm. Stat..

[26] Stratford P.W., Binkley J., Solomon P., Finch E., Gill C., Moreland J. (1996). Defining the minimum level of detectable change for the Roland-Morris questionnaire. Phys. Ther..

[27] Head H. (1926). Aphasia and Kindred Disorders of Speech.

[28] Bleiberg J., Garmoe W.S., Halpern E.L., Reeves D.L., Nadler J.D. (1997). Consistency of within-day and across-day performance after mild brain injury. Neuropsychiatry Neuropsychol. Behav. Neurol..

