# Supplementary material for: Alternative Devices for Heart Rate Variability Measures: A Comparative Test–Retest Reliability Study
Source: Behav Sci (Basel). 2021 May 2;11(5):68. doi: 10.3390/bs11050068 (PMC8147413; doi:10.3390/bs11050068)
Supplement: Supplementary file 1 [file behavsci-11-00068-s001.zip › behavsci-1090905-supplementary.pdf]

Supplement Killian, et al.

Alternative devices for heart rate variability measures: a comparative test-retest reliability study

Appendix

Determination of the Minimal Clinically Important Difference using a Global Rating Scale as a clinical anchor

Figures

Figure 1. Standard deviation of RR values for four devices in five behavioral conditions (SDNN)
Figure 2. Root mean square of successive difference values for four devices in five behavioral conditions (RMSSD)
Figure 3. Standard deviation of instantaneous heart values for four devices in five behavioral conditions (STD HR)
Figure 4. Mean heart rate values for four devices in five behavioral conditions (Mean HR)
Figure 5. Low frequency to high frequency ratio values for four devices in five behavioral conditions (LF/HF)

Tables

Table 1. Operational reliability of the four devices
Table 2. Numerical values of HRV measures
Table 3. Minimal detectable differences with confidence intervals (Detailed Specification)
Table 4. Intraclass correlation coefficients
Table 5. Standard error of measurement
Table 6. Bland-Altman limits of agreement (Detailed Specification)

Determination of the Minimal Clinically Important Difference using a Global Rating Scale as a clinical anchor

A. A measure of heart rate variability is obtained before and after treatment.
B. A GRS assessment is obtained from each patient after treatment to determine the patient's perception of clinical change. A commonly used GRS is a 15-point scale where -7="Much Worse", 0="No Change" and +7="Much Better".
C. Declare a 3-point change on the GRS ("Somewhat Better" or "Somewhat Worse") to be the minimal clinically important difference.
D. Use the 3-point change criterion to identify the subset of "minimal changers" within the patient population and determine the change in the HRV measure for each minimal changer.
E. The Minimal Clinically Important Difference is the average change (absolute value) observed in the minimal changer subpopulation.

Setting the minimal change criterion to three points on the GRS is a matter of clinical judgment. If the change criterion is increased, for example to five ("Quite a Bit Better" and "Quite a Bit Worse"), the Minimal Clinically Important Difference will increase. In addition to the Global Rating Scale, which is an subjective anchor, possible objective anchors include medication use and health care system utilization. Variants of anchor-based methods include within-patient score change, between-patients score change, sensitivity and specificity-based designs and social comparison designs [29].

Figure 1. **SDNN**

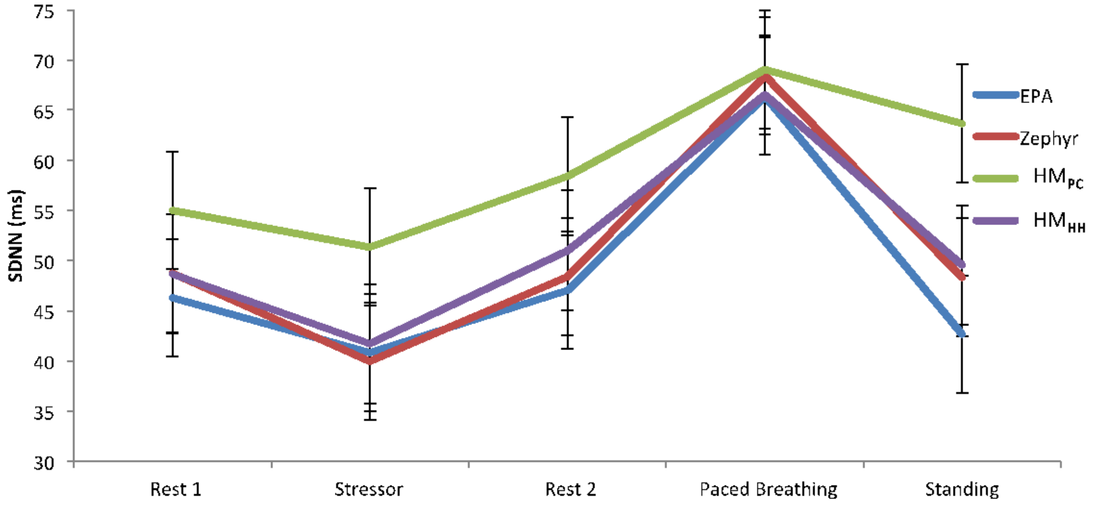

Figure 1. Standard Deviation of RR values (SDNN)**:** (a) When aggregating data across both sessions, there was a main effect of segment, indicating a general decrease in SDNN from initial rest, in response to the stressor, followed by a general increase during the recovery portion of the paradigm and then a slight decrease in SDNN during the standing segment (F=26.02, p<.001); (b) There appeared to be a main effect of device, such that HM_{PC} tended to display greater SDNN values compared to all other devices (F=9.32, p<.001), with post-hoc analyses revealing higher SDNN value at standing for the HM_{PC} device compared to the EPA6 device; and (c) when examining the interaction between device and segment all 4 devices tended to vary in a similar pattern throughout the session (F=0.75, p=.70). Data from a linear mixed model with two fixed factors (device, segment) and a random subject effect to calculate the difference between devices and between sessions.

Figure 2. **RMSSD**

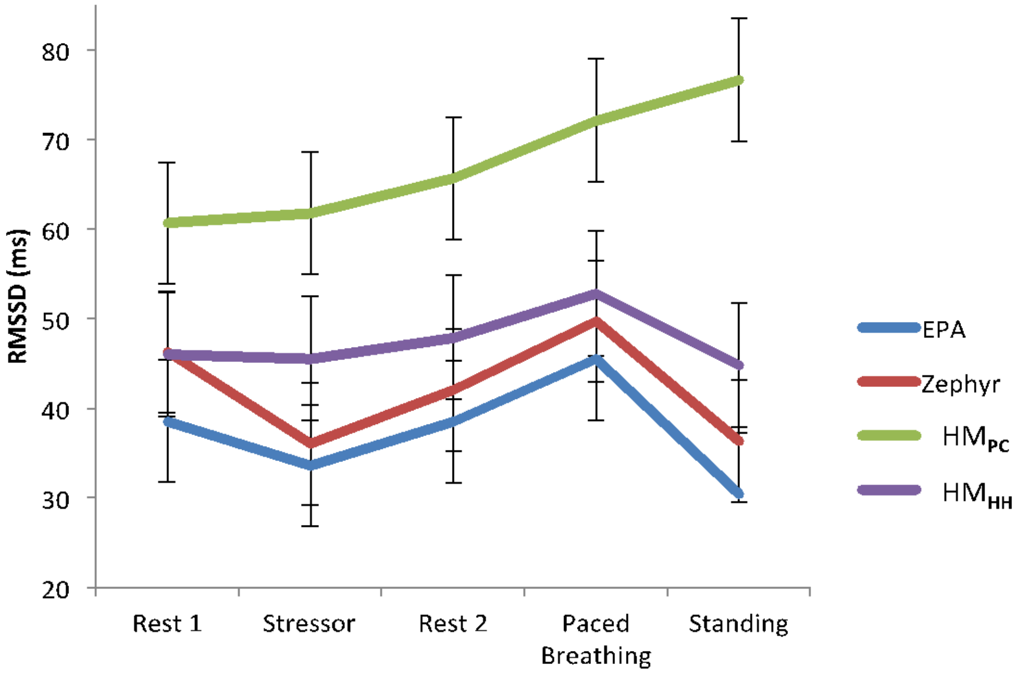

Figure 2. Root mean square of successive differences values (RMSSD): (a) When aggregating data across both sessions, there was a main effect of segment, indicating a general decrease in RMSSD from initial rest, in response to the stressor, followed by a general increase during the recovery portion of the paradigm and then a slight decrease in RMSSD during the standing segment (F=2.97, p=.02); (b) There appeared to be a main effect of device, such that HM_{PC} tended to display greater RMSSD values compared to all other devices (F=41.76, p<.001), with post-hoc analyses revealing higher RMSSD value at each time segment for the HM_{PC} device compared to EPA6; and (c) when examining the interaction between device and segment all 4 devices tended to vary in a similar pattern throughout the session (F=1.00, p=.45). Data from a linear mixed model with two fixed factors (device, segment) and a random subject effect to calculate the difference between devices and between sessions.

Figure 3. **STD HR**

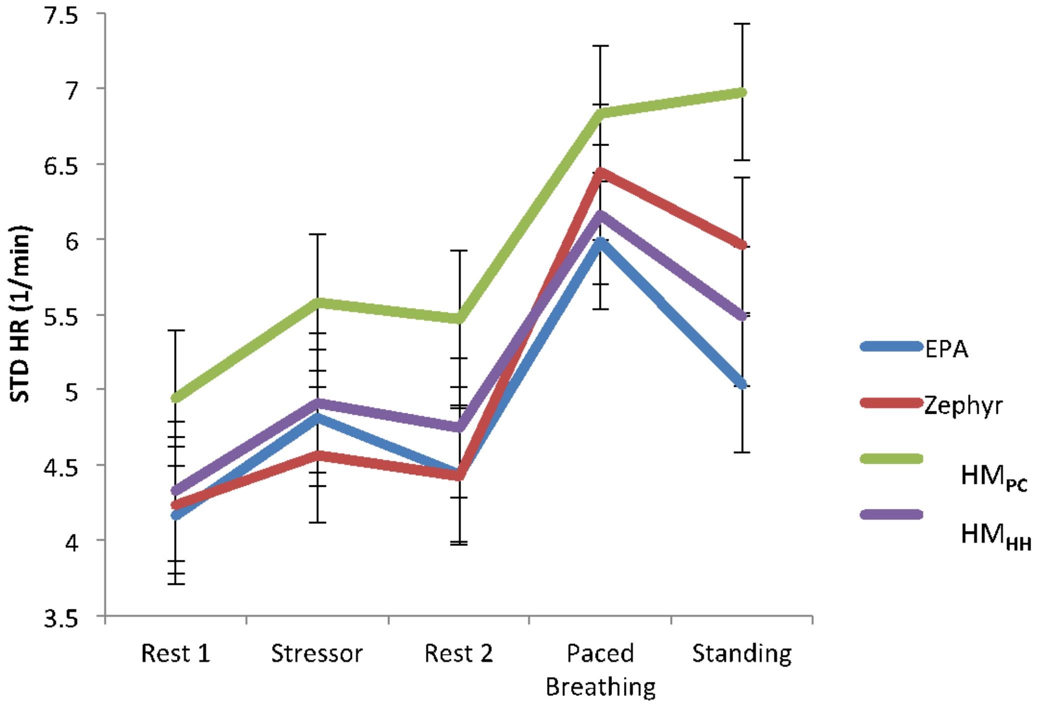

Figure 3. Standard deviation of instantaneous heart values (STD HR): (a) When aggregating data across both sessions, there was a main effect of segment, indicating a general increase in STD HR from initial rest, in response to the stressor, followed by a general decrease during the recovery portion of the paradigm and then a slight decrease in STD HR during the standing segment (F=19.62, p<.001); (b) There appeared to be a main effect of device, such that HM_{PC} tended to display greater STD HR values compared to all other devices (F=8.48, p<.001), with post-hoc analyses revealing higher STD HR value at standing for the HM_{PC} device compared to EPA6; and (c) when examining the interaction between device and segment all 4 devices tended to vary in a similar pattern throughout the session (F=.53, p=.90). Data from a linear mixed model with two fixed factors (device, segment) and a random subject effect to calculate the difference between devices and between sessions.

Figure 4. **Mean HR**

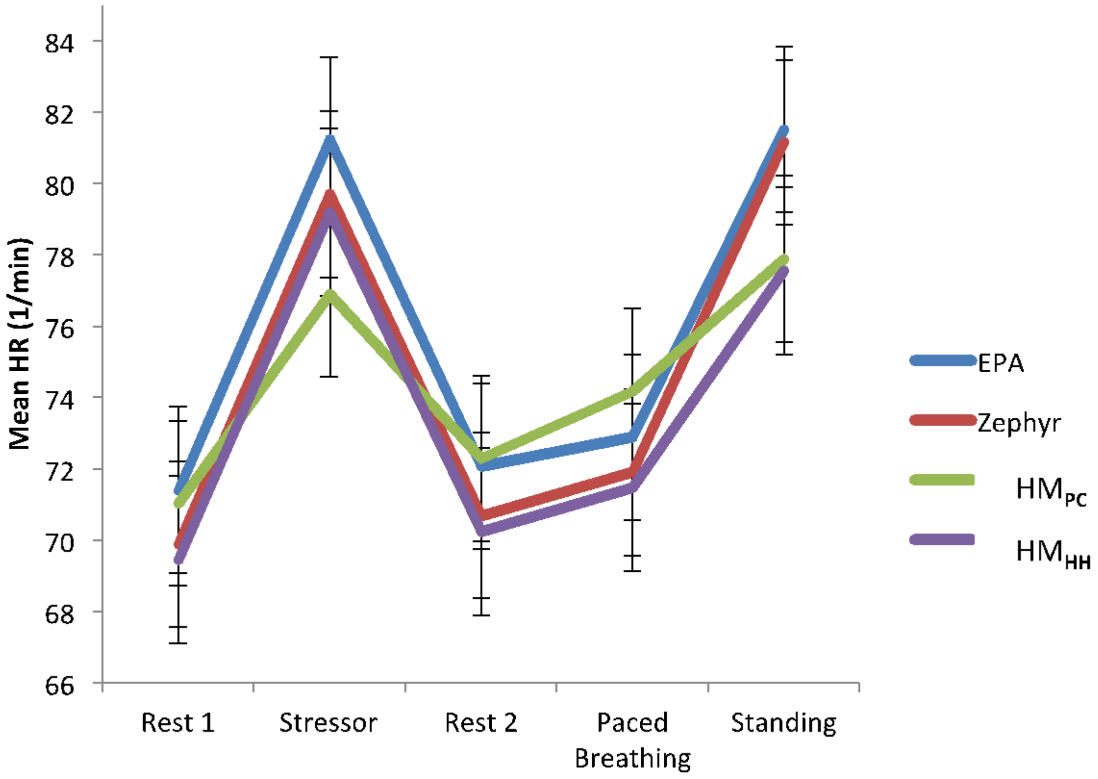

Figure 4. Mean heart rate values (Mean HR): (a) When aggregating data across both sessions, there was a main effect of segment, indicating a general increase in Mean HR from initial rest, in response to the stressor, followed by a general decrease during the recovery portion of the paradigm and then a slight increase in Mean HR during the standing segment (F=62.04, p<.001); (b) There appeared to be a main effect of device, such that HM_{HH} tended to display lower Mean HR values compared to the EPA6 device (F=3.32, p=.02); and (c) when examining the interaction between device and segment all 4 devices tended to vary in a similar pattern throughout the session (F=1.32, p=.20). Data from a linear mixed model with two fixed factors (device, segment) and a random subject effect to calculate the difference between devices and between sessions.

Figure 5. **Low Frequency / High Frequency (LF/HF)**

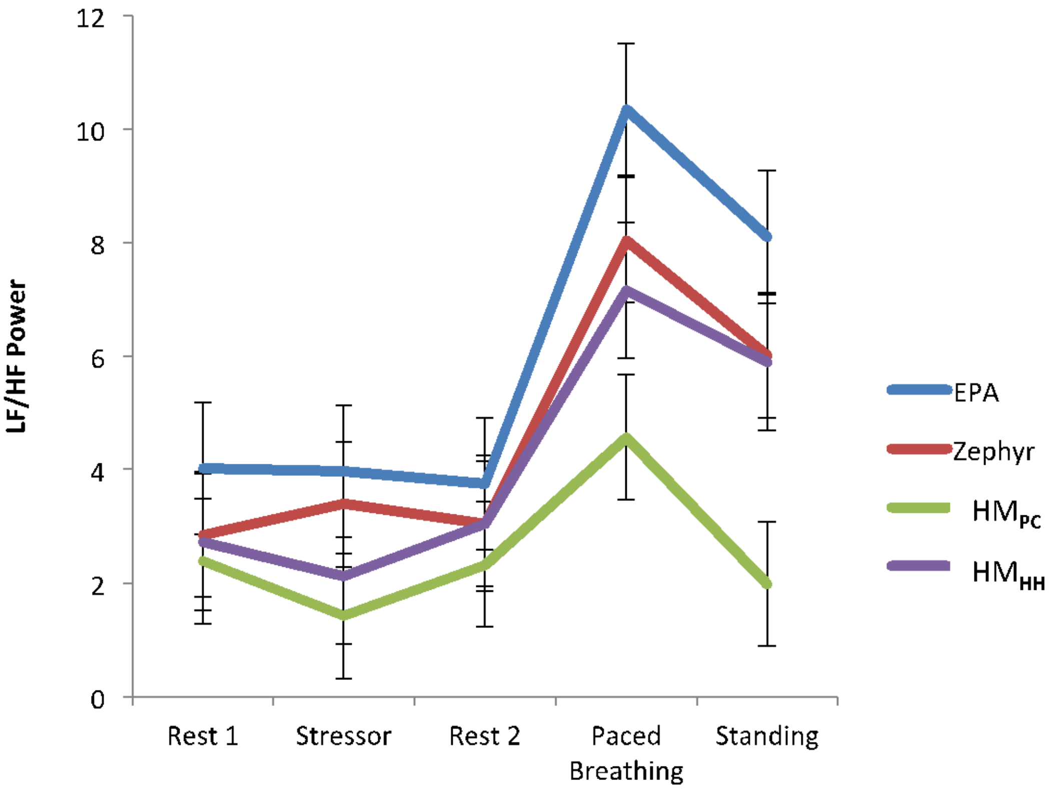

Figure 5. Low frequency to high frequency ratio values (LF/HF): (a) When aggregating data across both sessions, there was a main effect of segment, indicating a general increase in LF/HF from the stressor to the paced breathing portion of the paradigm and then a slight decrease in LF/HF during the standing segment (F=17.64, p<.001); (b) There appeared to be a main effect of device, such that HM$_{PC}$ tended to display lower LF/HF values compared to all other devices (F=10.65, p<.001) with post-hoc analyses revealing that at standing, HM$_{PC}$ displayed lower LF/HF compared to EPA6; and (c) when examining the interaction between device and segment all 4 devices tended to vary in a similar pattern throughout the session (F=1.01, p=.44). Data from a linear mixed model with two fixed factors (device, segment) and a random subject effect to calculate the difference between devices and between sessions.

Table 1. Operational reliability of the four devices

Device Operational Reliability expressed numerically as probability of successful operations (p_s) and probability of operational failures (p_f) based upon observed recordings of 140 time segments. ($p_s + p_f = 1$)

Device	Successful Operations	Probability of success	Probability of failure
EPA6	140/140	1.0000	0.0000
BioPatch	140/140	1.0000	0.0000
HM_{PC}	113/140	0.8071	0.1929
HM_{HH}	125/140	0.8929	0.1071

Table 2. Numerical Values of Heart Rate Variability Measures

Mean numerical values of six measures of heart rate variability in five conditions (Rest 1, Stressor, Rest 2, Paced Breathing, Standing) for each of four devices (EPA6, BioPatch, HM_{PC}, HM_{HH}). The numerical results present the mean values and the standard deviations of those means. The six measures are: mean RR interval (RR_{Mean}), standard deviation of RR intervals (SDNN), mean heart rate (HR_{Mean}), standard deviation of heart rate (STD HR), root mean square of successive differences (RMSSD) and the ratio of low frequency to high frequency spectral power (LF/HF Power), HF power, LF power (mean±standard deviation)

	**EPA6**	**BioPatch**	**HM_{PC}**	**HM_{HH}**
RR_{Mean} (ms) Rest 1	859.00 ± 108.19	876.91 ± 104.78	860.18 ± 87.87	896.20 ± 91.65
RR_{Mean} (ms) Stressor	754.23 ± 91.46	768.49 ± 85.92	794.14 ± 80.49	787.16 ± 87.30
RR_{Mean} (ms) Rest 2	849.80 ± 97.23	866.55 ± 98.11	846.10 ± 86.07	886.59 ± 87.66
RR_{Mean} (ms) Paced Breath	846.69 ± 114.02	858.76 ± 111.53	827.68 ± 85.66	878.04 ± 102.99
RR_{Mean} (ms) Standing	750.38 ± 78.84	755.11 ± 82.62	784.05 ± 57.59	800.35 ± 69.46
	**EPA6**	**BioPatch**	**HM_{PC}**	**HM_{HH}**
SDNN (ms) Rest 1	46.30 ± 26.17	48.75 ± 26.84	55.03 ± 26.49	50.20 ± 24.45
SDNN (ms) Stressor	40.81 ± 22.55	39.97 ± 18.76	51.34 ± 18.57	43.22 ± 14.02
SDNN (ms) Rest 2	47.06 ± 22.76	48.41 ± 22.80	58.38 ± 18.73	52.54 ± 23.48
SDNN (ms) Paced Breath	66.38 ± 27.98	68.41 ± 27.11	69.05 ± 16.19	68.04 ± 24.60
SDNN (ms) Standing	42.64 ± 15.86	48.33 ± 20.39	63.68 ± 15.26	51.08 ± 15.11
	**EPA6**	**BioPatch**	**HM_{PC}**	**HM_{HH}**
HR_{Mean} (1/min) Rest 1	71.40 ± 9.27	69.89 ± 8.36	71.03 ± 7.52	68.08 ± 6.64
HR_{Mean} (1/min) Stressor	81.22 ± 9.69	79.69 ± 8.99	76.90 ± 7.96	77.82 ± 8.90
HR_{Mean} (1/min) Rest 2	72.08 ± 8.56	70.69 ± 8.29	72.29 ± 7.68	68.89 ± 6.72
HR_{Mean} (1/min) Paced Breath	72.88 ± 10.45	71.89 ± 9.72	74.16 ± 8.39	70.11 ± 8.53
HR_{Mean} (1/min) Standing	81.51 ± 8.46	81.15 ± 8.85	77.89 ± 5.91	76.18 ± 6.72

Table 2. Continued Numerical Values of Heart Rate Variability Measures

	**EPA6**	**BioPatch**	**HM_{PC}**	**HM_{HH}**
STD HR (1/min) Rest 1	4.166 ± 1.502	4.236 ± 1.304	4.944 ± 1.581	4.354 ± 1.619
STD HR (1/min) Stressor	4.815 ± 2.095	4.569 ± 1.352	5.578 ± 1.720	4.936 ± 1.321
STD HR (1/min) Rest 2	4.442 ± 1.268	4.426 ± 1.190	5.471 ± 1.114	4.773 ± 1.831
STD HR (1/min) Paced Breath	5.986 ± 2.103	6.442 ± 2.064	6.831 ± 1.822	6.187 ± 1.944
STD HR (1/min) Standing	5.033 ± 1.554	5.958 ± 2.285	6.971 ± 2.076	5.512 ± 1.419
	**EPA6**	**BioPatch**	**HM_{PC}**	**HM_{HH}**
RMSSD (ms) Rest1	38.55 ± 26.09	46.27 ± 32.72	60.68 ± 37.85	47.22 ± 23.68
RMSSD (ms) Stressor	33.59 ± 25.53	36.04 ± 24.92	61.78 ± 27.74	46.74 ± 17.18
RMSSD (ms) Rest 2	38.53 ± 24.43	42.04 ± 24.44	65.69 ± 29.19	49.08 ± 24.52
RMSSD (ms) Paced Breath	45.46 ± 26.29	49.74 ± 24.54	72.12 ± 23.20	53.99 ± 23.91
RMSSD (ms) Standing	30.42 ± 19.82	36.35 ± 21.76	76.63 ± 24.78	45.99 ± 18.68
	**EPA6**	**BioPatch**	**HM_{PC}**	**HM_{HH}**
LF/HF Power Rest 1	4.021± 3.626	2.851 ± 2.151	2.392 ± 2.960	2.770 ± 2.456
LF/HF Power Stressor	3.969 ± 2.953	3.394 ± 2.552	1.422 ± 0.824	2.175 ± 1.890
LF/HF Power Rest 2	3.754 ± 3.048	3.043 ± 1.995	2.329 ± 2.178	3.106 ± 2.400
LF/HF Power Paced Breath	10.348 ± 7.581	8.043 ± 6.079	4.568 ± 5.451	7.213 ± 5.949
LF/HF Power Standing	8.101 ± 8.427	6.016 ± 4.724	1.986 ± 2.626	5.946 ± 6.793

Table 2. Continued Numerical Values of Heart Rate Variability Measures

	EPA6	BioPatch	HM_{PC}	HM_{HH}
HF Power Rest 1	30.964 ±20.824	34.039 ±18.954	48.393 ±21.396	38.110 ±18.993
HF Power Stressor	26.447 ±14.026	28.596 ±14.637	48.491 ±15.802	36.091 ±16.259
HF Power Rest 2	30.542 ±19.558	31.449 ±16.955	46.899 ±21.156	33.576 ±18.952
HF Power Paced Breath	16.424 ±14.533	17.821 ±12.902	44.415 ±21.593	22.295 ±17.870
HF Power Standing	22.203 ±18.077	22.109 ±14.601	50.051 ±15.598	24.081 ±15.026

	EPA6	BioPatch	HM_{PC}	HM_{HH}
LF Power Rest 1	63.572 ±20.261	60.118 ±18.498	47.138 ±20.106	55.998 ±18.548
LF Power Stressor	67.269 ±13.179	64.718 ±13.730	47.600 ±15.116	58.050 ±15.358
LF Power Rest 2	62.908 ±19.153	63.069 ±16.081	48.939 ±20.401	59.706 ±17.103
LF Power Paced Breath	81.435 ±15.569	79.269 ±14.794	52.738 ±22.290	74.987 ±19.324
LF Power Standing	73.811 ±18.998	73.030 ±16.814	46.795 ±15.411	70.204 15.895

Table 3. Minimal Detectable Difference with Confidence Intervals

	EPA6	BioPatch	HM_{PC}	HM_{HH}
RR_{Mean} (ms) Rest1	137.21 [80.66, 219.13]	146.35 [86.74, 229.34]	137.00 [75.56, 220.89]	126.23 [66.05, 216.11]
RR_{Mean} (ms) Stressor	136.79 [81.64, 211.11]	132.19 79.15, 202.62]	105.73 [57.24, 178.43]	140.14 [74.71, 229.03]
RR_{Mean} (ms) Rest 2	158.55 [95.62, 239.43	155.00 [93.11, 236.06]	158.07 [89.48, 241.20]	138.83 [73.87, 227.90]
RR_{Mean} (ms) Paced Breath1	108.51 [62.75, 180.59]	108.28 [62.67, 179.80]	136.15 [75.29, 218.24]	120.37 [61.10, 214.41]
RR_{Mean} (ms) Standing1	159.47 [99.82, 225.12]	117.60 [69.83, 183.54]	149.06 [93.67, 194.44]	122.53 [59.89, 203.61]
	**EPA6**	**BioPatch**	**HM_{PC}**	**HH_{HH}**
SDNN (ms) Rest 1	29.83 [17.41, 48.49]	72.08 [49.48, 90.12]	26.63 [14.14, 47.29]	28.71 [14.82, 51.09]
SDNN (ms) Stressor	56.29 [37.47, 72.89]	23.79 [13.98, 37.99]	17.07 [9.04, 30.69]	17.14 [8.87, 30.23]
SDNN (ms) Rest 2	20.17 [11.63, 33.82]	21.14 [12.21, 35.28]	35.51 [20.23, 53.53]	21.71 [11.05, 40.30]
SDNN (ms) Paced Breath	33.04 [19.33, 53.41]	32.01 [18.72, 51.75]	27.67 [15.47, 43.32]	32.20 [16.76, 55.87]
SDNN (ms) Standing	16.60 [9.64, 27.31]	22.63 [13.19, 36.94]	26.21 [14.67, 40.97]	19.61 [9.18, 36.55]
	**EPA6**	**BioPatch**	**HM_{PC}**	**HM_{HH}**
HR_{Mean} (1/min) Rest 1	11.05 [6.46, 17.83]	11.73 [6.95, 18.36]	10.84 [5.92, 17.87]	9.87 [5.21, 16.54]
HR_{Mean} (1/min) Stressor	16.96 [10.33, 25.10]	16.42 [10.08, 23.99]	10.64 [5.77, 17.88]	17.65 [9.79, 26.59]
HR_{Mean} (1/min) Rest 2	13.97 [8.43, 21.10]	12.91 [7.74, 19.73]	13.51 [7.59, 20.94]	11.84 [6.41, 18.71]
HR_{Mean} (1/min) Paced Breath	10.14 [5.87, 16.84]	10.15 [5.89, 16.70]	12.36 [6.77, 20.26]	10.72 [5.56, 18.78]
HR_{Mean} (1/min) Stdanding	17.14 [10.73, 24.18]	12.24 [7.25, 19.22]	15.34 [9.65, 19.98]	12.67 [6.28, 20.41]

Table 3. Continued Minimal Detectable Difference with Confidence Intervals

	EPA6	BioPatch	HM_{PC}	HM_{HH}
STD HR (ms) Rest1	2.101 [1.245, 3.291]	1.515 [0.885, 2.456]	2.860 [1.615, 4.389]	2.085 [1.084, 3.632]
STD HR (ms) Stressor	5.163 [3.420, 6.725]	2.288 [1.387, 3.421]	2.201 [1.189, 3.736]	2.101 [1.118, 3.444]
STD HR (ms) Rest 2	1.710 [1.010, 2.699]	1.863 [1.118, 2.844]	3.092 [2.015, 3.878]	2.989 [1.597, 4.857]
STD HR (ms) Paced Breath1	3.172 [1.895, 4.886]	5.338 [3.603, 6.798]	3.276 [1.847, 5.038]	2.795 [1.469, 4.728]
STD HR (ms) Standing1	2.269 [1.351, 3.521]	4.862 [3.080, 6.735]	3.789 [2.142, 5.795]	2.291 [1.103, 3.954]
	**EPA6**	**BioPatch**	**HM_{PC}**	**HH_{HH}**
RMSSD (ms) Rest 1	31.15 [18.23, 50.26]	84.95 [57.44, 107.98]	30.96 [16.32, 56.44]	45.44 [25.01, 69.41]
RMSSD (ms) Stressor	53.15 [33.49, 74.24]	44.51 [27.21, 65.49]	35.92 [19.42, 60.82]	28.80 [15.46, 46.36]
RMSSD (ms) Rest 2	33.27 [19.68, 52.42]	37.40 [22.38, 57.41]	47.51 [26.36, 75.59]	39.55 [21.10, 64.53]
RMSSD (ms) Paced Breath	36.11 [21.37, 56.79]	27.22 [15.86, 44.43]	34.09 [18.67, 55.91]	25.95 [13.32, 46.91]
RMSSD (ms) Standing	33.98 [20.64, 50.61]	29.40 [17.37, 46.38]	42.19 [23.57, 66.14]	27.76 [13.20, 49.95]
	**EPA6**	**BioPatch**	**HM_{PC}**	**HM_{HH}**
LF/HF Power Rest 1	3.396 [1.962, 5.661]	2.384 [1.389, 3.891]	2.841 [1.507, 5.079]	3.635 [1.917, 6.096]
LF/HF Power Stressor	5.044 [3.062, 7.519]	3.592 [2.131, 5.619]	2.217 [1.421, 2.831]	2.442 [1.270, 4.251]
LF/HF Power Rest 2	3.754 [2.202, 6.026]	4.016 [2.511, 5.678]	4.430 [2.564, 6.491]	2.902 [1.501, 5.131]
LF/HF Power Paced Breath	14.54 [9.002, 20.909]	13.955 [9.028, 18.751]	7.524 [4.092, 12.545]	13.785 [8.001, 19.283]
LF/HF Power Standing	7.571 [4.368, 12.676]	9.210 [5.721, 13.171]	7.467 [4.938, 9.236]	19.062 [11.102, 24.406]

Table 3. Continued Minimal Detectable Difference with Confidence Intervals

	EPA6	BioPatch	HM_{PC}	HM_{HH}
HF Power Rest 1	28.24 [5.86, 39.50]	24.10 [3.97, 33.85]	21.16 [0.00, 31.11]	32.74 [0.00, 46.92]
HF Power Stressor	23.60 [7.94, 32.42]	20.79 [4.90, 28.99]	35.43 [14.43, 47.98]	20.26 [0.00, 29.87]
HF Power Rest 2	20.71 [0.00, 29.35]	20.80 [2.90, 29.27]	34.83 [0.00, 49.52]	26.63 [0.00, 38.94]
HF Power Paced Breath	20.74 [4.95, 28.91]	22.14 [7.70, 30.35]	33.93 [0.00, 48.47]	42.49 [17.73, 57.42]
HF Power Standing	35.58 [15.39, 47.91]	19.60 [3.94, 27.44]	43.78 [27.55, 55.45]	26.51 [0.00, 39.15]

	EPA6	BioPatch	HM_{PC}	HM_{HH}
LF Power Rest 1	26.73 [5.05, 37.46]	20.26 [0.00, 28.68]	21.14 [0.00, 31.00]	32.63 [0.00, 46.65]
LF Power Stressor	24.75 [9.97, 33.55]	23.49 [8.14, 32.21]	32.86 [12.37, 44.80]	23.94 [0.00, 34.69]
LF Power Rest 2	20.60 [0.00, 29.17]	22.18 [4.82, 30.99]	32.42 [0.00, 46.25]	24.44 [0.00, 35.69]
LF Power Paced Breath	37.56 [9.61, 52.25]	30.10 [13.65, 40.32]	33.38 [0.00, 47.89]	46.87 [20.61, 62.99]
LF Power Standing	35.86 [14.56, 48.57]	24.50 [6.15, 34.10]	43.49 [27.60, 54.96]	30.52 [0.00, 44.50]

Table 4. Intraclass correlation coefficients

Intraclass correlation coefficients (ICC (2,1), Shrout and Fleiss, 1979) for six measures of heart rate variability (Mean RR, SDNN, Mean HR, STD HR, RMSSD, LF/HF Power), in five conditions (Rest 1, Stressor, Rest 2, Paced Breathing, Standing), for each of four devices (EPA6, BioPatch, HM_{PC}, HM_{HH}). Numerical results report the intraclass correlation coefficient, and the corresponding 95% confidence intervals.

	**EPA6**	**Biopatch**	**HM_{PC}**	**HM_{HH}**
RR_{Mean} (ms) Rest 1	0.7907 [0.4661, 0.9277]	0.7461 [0.3764, 0.9108]	0.6836 [0.1776, 0.9038]	0.7531 [0.2763, 0.9324]
RR_{Mean} (ms) Stressor	0.7088 [0.3065, 0.8963]	0.6919 [0.2761, 0.8895]	0.7755 [0.3605, 0.9342]	0.6646 [0.1042, 0.9047]
RR_{Mean} (ms) Rest 2	0.6540 [0.2108, 0.8741]	0.6751 [0.2468, 0.8828]	0.5610 [-0.0222, 0.8593]	0.6735 [0.1202, 0.9076]
RR_{Mean} (ms) Paced Breath	0.8821 [0.6735, 0.9606]	0.8773 [0.6617, 0.9589]	0.6712 [0.1552, 0.8995]	0.8222 [0.4359, 0.9527]
RR_{Mean} (ms) Standing	0.4675 [-0.0611, 0.7914]	0.7363 [0.3576, 0.9070]	0.1281 [-0.4836, 0.6557]	0.5950 [-0.1183, 0.9032]
	**EPA6**	**Biopatch**	**HM_{PC}**	**HM_{HH}**
SDNN (ms) Rest 1	0.8309 [0.5532, 0.9424]	0.0614 [-0.4673, 0.5578]	0.8684 [0.5851, 0.9628]	0.8205 [0.4317, 0.9522]
SDNN (ms) Stressor	0.1890 [-0.3599, 0.6408]	0.7907 [0.4662, 0.9277]	0.8901 [0.6445, 0.9692]	0.8055 [0.3950, 0.9479]
SDNN (ms) Rest 2	0.8977 [0.7125, 0.9660]	0.8882 [0.6884, 0.9627]	0.5322 [-0.0631, 0.8482]	0.8887 [0.6165, 0.9711]
SDNN (ms) Paced Breath	0.8185 [0.5256, 0.9379]	0.8185 [0.5257, 0.9379]	0.6196 [0.0678, 0.8811]	0.7772 [0.3290, 0.9396]
SDNN (ms) Standing	0.8574 [0.6140, 0.9519]	0.8397 [0.5731, 0.9456]	0.6159 [0.0619, 0.8798]	0.7810 [0.2389, 0.9519]

Table 4. Continued: Intraclass correlation coefficients

	EPA6	Biopatch	HMPC	HMHH
HR_{Mean} (1/min) Rest 1	0.8152 [0.5185, 0.9367]	0.7439 [0.3722, 0.9099]	0.7296 [0.2650, 0.9193]	0.7127 [0.1937, 0.9200]
HR_{Mean} (1/min) Stressor	0.6019 [0.1272, 0.8522]	0.5656 [0.0728, 0.8364]	0.7677 [0.3437, 0.9317]	0.4883 [-0.1612, 0.8426]
HR_{Mean} (1/min) Rest 2	0.6536 [0.2103, 0.8740]	0.6843 [0.2628, 0.8865]	0.5966 [0.0314, 0.8727]	0.5957 [-0.0100, 0.8816]
HR_{Mean} (1/min) Paced Breath	0.8774 [0.6619, 0.9589]	0.8581 [0.6157, 0.9521]	0.7173 [0.2408, 0.9152]	0.7944 [0.3687, 0.9447]
HR_{Mean} (1/min) Standing	0.4657 [-0.0635, 0.7905]	0.7511 [0.3862, 0.9127]	0.1233 [-0.4873, 0.6529]	0.5371 [-0.2013, 0.8863]
	**EPA6**	**Biopatch**	**HM_{PC}**	**HM_{HH}**
STD HR Rest 1	0.7455 [0.3753, 0.9106]	0.8244 [0.5387, 0.9401]	0.5739 [-0.0031, 0.8642]	0.7843 [0.3452, 0.9417]
STD HR Stressor	0.2093 [-0.3414, 0.6530]	0.6272 [0.1671, 0.8630]	0.7869 [0.3857, 0.9378]	0.6709 [0.1154, 0.9067]
STD HR Rest 2	0.7636 [0.4109, 0.9175]	0.6807 [0.2564, 0.8850]	-0.0030 [-0.5780, 0.5739]	0.6534 [0.0845, 0.9010]
STD HR Paced Breath	0.7037 [0.2973, 0.8943]	0.1295 [-0.4119, 0.6033]	0.5794 [0.0052, 0.8663]	0.7309 [0.2301, 0.9256]
STD HR Standing	0.7225 [0.3317, 0.9017]	0.4106 [-0.1310, 0.7635]	0.5665 [-0.0141, 0.8614]	0.6608 [-0.0099, 0.9214]

Table 4. Continued: Intraclass correlation coefficients

	EPA6	Biopatch	HM_{PC}	HM_{HH}
RMSSD (ms) Rest1	0.8145 [0.5170, 0.9365]	0.1224 [-0.4178, 0.5987]	0.9129 [0.7106, 0.9758]	0.5205 [-0.1188, 0.8547]
RMSSD (ms) Stressor	0.4361 [-0.1004, 0.7761]	0.5848 [0.1013, 0.8449]	0.7818 [0.3743, 0.9362]	0.6340 [0.0517, 0.8946]
RMSSD (ms) Rest 2	0.7587 [0.4011, 0.9156]	0.6953 [0.2822, 0.8909]	0.6553 [0.1274, 0.8939]	0.6613 [0.0984, 0.9036]
RMSSD (ms) Paced Breath	0.7544 [0.3926, 0.9140]	0.8398 [0.5734, 0.9456]	0.7188 [0.2438, 0.9157]	0.8467 [0.4989, 0.9596]
RMSSD (ms) Standing	0.6173 [0.1513, 0.8588]	0.7624 [0.4085, 0.9170]	0.6227 [0.0728, 0.8822]	0.7124 [0.0875, 0.9349]

	EPA6	Biopatch	HM_{PC}	HM_{HH}
LF/HF Power Rest 1	0.8858 [0.6827, 0.9619]	0.8401 [0.5739, 0.9457]	0.8802 [0.6169, 0.9663]	0.7148 [0.1979, 0.9207]
LF/HF Power Stressor	0.6204 [0.1562, 0.8601]	0.7421 [0.3688, 0.9093]	0.0573 [-0.5364, 0.6131]	0.7827 [0.3416, 0.9412]
LF/HF Power Rest 2	0.8026 [0.4913, 0.9321]	0.4725 [-0.0547, 0.7937]	0.4618 [-0.1556, 0.8197]	0.8097 [0.4052, 0.9491]
LF/HF Power Paced Breath	0.5212 [0.0098, 0.8165]	0.3141 [-0.2384, 0.7130]	0.7521 [0.3107, 0.9267]	0.3011 [-0.3676, 0.7646]
LF/HF Power Standing	0.8950 [0.7055, 0.9650]	0.5053 [-0.0117, 0.8091]	-0.0525 [-0.6100, 0.5398]	-0.0248 [-0.6799, 0.6524]

Table 4. Continued: Intraclass correlation coefficients

	EPA6	Biopatch	HM_{PC}	HM_{HH}
HF Power Rest 1	0.7607 [0.5317, 0.9897]	0.7896 [0.5849, 0.9943]	0.8727 [0.7248, 1.020]	0.6132 [0.2056, 1.021]
HF Power Stressor	0.6315 [0.3047, 0.9583]	0.7375 [0.4896, 0.9854]	0.3458 [-0.200, 0.8915]	0.7979 [0.5606, 1.035]
HF Power Rest 2	0.8540 [0.7068, 1.001]	0.8042 [0.6122, 0.9962]	0.6472 [0.2870, 1.007]	0.7430 [0.4504, 1.036]
HF Power Paced Breath	0.7349 [0.4849, 0.9849]	0.6167 [0.2798, 0.9536]	0.6787 [0.3443, 1.013]	0.2642 [-0.3436, 0.8719]
HF Power Standing	0.4957 [0.0857, 0.9057]	0.7654 [0.5403, 0.9905]	-0.0253 [-0.6449, 0.5939]	0.5949 [0.1163, 1.074]

	EPA6	Biopatch	HM_{PC}	HM_{HH}
LF Power Rest 1	0.7735 [0.5551, 0.9919]	0.8438 [0.6872, 1.000]	0.8561 [0.6906, 1.022]	0.5972 [0.1768, 1.018]
LF Power Stressor	0.5409 [0.1563, 0.9255]	0.6189 [0.2835, 0.9543]	0.3850 [-0.1430, 0.9129]	0.6838 [0.3359, 1.032]
LF Power Rest 2	0.8495 [0.6981, 1.001]	0.7525 [0.5167, 0.9883]	0.6714 [0.3310, 1.012]	0.7343 [0.4332, 1.035]
LF Power Paced Breath	0.7191 [0.4565, 0.9816]	0.4613 [0.0333, 0.8892]	0.7081 [0.3991, 1.017]	0.2344 [-0.3830, 0.8519]
LF Power Standing	0.5364 [0.1493, 0.9236]	0.7236 [0.4646, 0.9826]	-0.0366 [-0.6556, 0.5824]	0.5202 [-0.0202, 1.061]

Table 5. Standard Error of Measurement

	**EPA6**	**BioPatch**	**HM_{PC}**	**HM_{HH}**
RR_{Mean} (ms)<br>Rest1	49.50<br>[29.10, 79.05]	52.80<br>[31.29, 82.74]	49.42<br>[27.26, 79.69]	45.54<br>[23.83, 77.97]
RR_{Mean} (ms)<br>Stressor	49.35<br>[29.45, 76.16]	47.69<br>[28.56, 73.10]	38.14<br>[20.65, 64.37]	50.56<br>[26.95, 82.63]
RR_{Mean} (ms)<br>Rest 2	57.20<br>[34.50, 86.38]	55.92<br>[33.59, 85.15]	57.03<br>[32.28, 87.02]	50.08<br>[26.65, 82.22]
RR_{Mean} (ms)<br>Paced Breath1	39.15<br>[22.64, 65.15]	39.06<br>[22.61, 64.87]	49.12<br>[27.16, 78.73]	43.43<br>[22.41, 77.35]
RR_{Mean} (ms)<br>Standing1	57.53<br>[36.01, 81.22]	42.43<br>[25.19, 66.22]	53.78<br>[33.79, 70.15]	44.21<br>[21.61, 73.46]
	**EPA6**	**BioPatch**	**HM_{PC}**	**HH_{HH}**
SDNN (ms)<br>Rest 1	10.76<br>[6.29, 17.49]	26.00<br>[17.85, 32.51]	9.61<br>[5.11, 17.06]	10.36<br>[5.35, 18.43]
SDNN (ms)<br>Stressor	20.31<br>[13.52, 26.30]	8.58<br>[5.04, 13.71]	6.16<br>[3.26, 11.07]	6.18<br>[3.20, 10.91]
SDNN (ms)<br>Rest 2	7.28<br>[4.20, 12.20]	7.63<br>[4.41, 12.73]	12.81<br>[7.30, 19.31]	7.83<br>[3.99, 14.54]
SDNN (ms)<br>Paced Breath	11.92<br>[6.97, 19.27]	11.55<br>[6.75, 18.67]	9.98<br>[5.58, 15.63]	11.62<br>[6.05, 20.15]
SDNN (ms)<br>Standing	5.99<br>[3.48, 9.85]	8.16<br>[4.76, 13.33]	9.46<br>[5.29, 14.78]	7.07<br>[3.31, 13.19]
	**EPA6**	**BioPatch**	**HM_{PC}**	**HM_{HH}**
HR_{Mean} (1/min)<br>Rest 1	3.99<br>[2.33, 6.43]	4.23<br>[2.51, 6.62]	3.91<br>[2.14, 6.45]	3.56<br>[1.88, 5.97]
HR_{Mean} (1/min)<br>Stressor	6.12<br>[3.73, 9.06]	5.92<br>[3.64, 8.66]	3.84<br>[2.08, 6.45]	6.37<br>[3.53, 9.59]
HR_{Mean} (1/min)<br>Rest 2	5.04<br>[3.04, 7.61]	4.66<br>[2.79, 7.12]	4.88<br>[2.74, 7.55]	4.27<br>[2.31, 6.75]
HR_{Mean} (1/min)<br>Paced Breath	3.66<br>[2.12, 6.08]	3.66<br>[2.13, 6.03]	4.46<br>[2.44, 7.31]	3.87<br>[2.01, 6.78]
HR_{Mean} (1/min)<br>Stdanding	6.18<br>[3.87, 8.72]	4.42<br>[2.61, 6.93]	5.53<br>[3.48, 7.21]	4.57<br>[2.27, 7.36]

Table 5. Continued. Standard Error of Measurement

	**EPA6**	**BioPatch**	**HM_{PC}**	**HM_{HH}**
STD HR (ms) Rest1	0.758 [0.449, 1.187]	0.547 [0.319, 0.886]	1.032 [0.583, 1.583]	0.752 [0.391, 1.310]
STD HR (ms) Stressor	1.863 [1.234, 2.426]	0.826 [5.501, 1.234]	0.794 [0.429, 1.348]	0.758 [0.404, 1.243]
STD HR (ms) Rest 2	0.617 [0.364, 0.974]	0.672 [0.403, 1.026]	1.115 [0.727, 1.399]	1.078 [0.576, 1.752]
STD HR (ms) Paced Breath1	1.145 [0.684, 1.763]	1.926 [1.300, 2.452]	1.182 [0.666, 1.818]	1.009 [0.530, 1.706]
STD HR (ms) Standing1	0.818 [0.487, 1.270]	1.754 [1.111, 2.430]	1.367 [0.773, 2.091]	0.827 [0.398, 1.426]
	**EPA6**	**BioPatch**	**HM_{PC}**	**HH_{HH}**
RMSSD (ms) Rest 1	11.24 [6.58, 18.13]	30.65 [20.72, 38.96]	11.17 [5.89, 20.36]	16.39 [9.02, 25.04]
RMSSD (ms) Stressor	19.17 [12.08, 26.78]	16.06 [9.82, 23.63]	12.96 [7.01, 21.94]	10.39 [5.58, 16.73]
RMSSD (ms) Rest 2	12.00 [7.09, 18.91]	13.49 [8.07, 20.71]	17.14 [9.51, 27.27]	14.27 [7.61, 23.28]
RMSSD (ms) Paced Breath	13.03 [7.71, 20.49]	9.82 [5.72, 16.03]	12.30 [6.74, 20.17]	9.36 [4.81, 16.92]
RMSSD (ms) Standing	12.26 [7.45, 18.26]	10.61 [6.27, 16.73]	15.22 [8.50, 23.86]	10.02 [4.76, 17.84]
	**EPA6**	**BioPatch**	**HM_{PC}**	**HM_{HH}**
LF/HF Power Rest 1	1.225 [0.708, 2.042]	0.860 [0.501, 1.404]	1.025 [0.544, 1.832]	1.312 [0.692, 2.199]
LF/HF Power Stressor	1.820 [1.105, 2.713]	1.296 [0.769, 2.027]	0.800 [0.513, 1.021]	0.881 [0.458, 1.534]
LF/HF Power Rest 2	1.354 [0.794, 2.174]	1.449 [0.906, 2.049]	1.598 [0.925, 2.342]	1.047 [0.541, 1.851]
LF/HF Power Paced Breath	5.246 [3.248, 7.543]	5.035 [3.257, 6.765]	2.714 [1.476, 4.526]	4.973 [2.886, 6.957]
LF/HF Power Standing	2.731 [1.576, 4.573]	3.323 [2.064, 4.752]	2.694 [1.781, 3.332]	6.877 [4.005, 8.805]

Table 5. Continued. Standard Error of Measurement

	EPA6	BioPatch	HMPC	HMHH
HF Power Rest 1	10.19 [2.11, 14.25]	8.69 [1.43, 12.21]	7.63 [0.00, 11.22]	11.81 [0.00, 16.93]
HF Power Stressor	8.51 [2.86, 11.70]	7.50 [1.77, 10.46]	12.78 [5.21, 17.31]	7.31 [0.00, 10.78]
HF Power Rest 2	7.47 [0.00, 10.59]	7.50 [1.05, 10.56]	12.57 [0.00, 17.86]	9.61 [0.00, 14.05]
HF Power Paced Breath	7.48 [1.79, 10.43]	7.99 [2.78, 10.95]	12.24 [0.00, 17.48]	15.33 [6.40, 20.71]
HF Power Standing	12.84 [5.55, 17.29]	7.07 [1.42, 9.90]	15.79 [9.94, 20.01]	9,56 [0.00, 14.13]

	EPA6	BioPatch	HMPC	HMHH
LF Power Rest 1	9.643 [1.023, 13.514]	7.311 [0.000, 10.346]	7.627 [0.000, 11.184]	11.772 [0.000, 16.829]
LF Power Stressor	8.930 [3.597, 12.105]	8.476 [2.935, 11.622]	11.854 [4.461, 16.161]	8.636 [0.000, 12.516]
LF Power Rest 2	7.430 [0.000, 10.524]	8.000 [1.739, 11.179]	11.695 [0.000, 16.686]	8.816 [0.000, 12.876]
LF Power Paced Breath	13.552 [3.468, 18.850]	10.858 [4.924, 14.546]	12.043 [0.000, 17.279]	16.908 [7.437, 22.725]
LF Power Standing	12.935 [5.251, 17.523]	8.840 [2.218, 12.303]	15.690 [9.959, 19.829]	11.010 [0.000, 16.055]

Table 6. Bland-Altman Limits of Agreement

Limits of Agreement and 95% Confidence Intervals of five measures of heart rate variability (Mean RR, SDNN, Mean HR, STD HR, RMSSD, LF/HF Power), in five conditions (Rest 1, Stressor, Rest 2, Paced Breathing, Standing), comparing three devices (BioPatch, $HM_{PC,}$ HM_{HH}) against the EPA6. Numerical results are: (1.) mean value of the inter-device difference and the standard deviation of that mean, (2.) the lower limit of agreement and the confidence interval of that limit of agreement, (3.) the upper limit of agreement and the confidence interval of that limit of agreement.

Device 1	Device 2	Segment	Measure	Mean Difference (SD)	Lower Limit of Agreement (95%CI)	Upper Limit of Agreement (95% CI)
EPA6	BioPatch	Rest 1	Mean RR	-18.808 (13.906)	-46.063 (-59.97, -32.157)	8.447 (-5.459, 22.354)
EPA6	BioPatch	Stressor	Mean RR	-14.704 (6.82)	-28.071 (-34.892, -21.251)	-1.336 (-8.156, 5.485)
EPA6	BioPatch	Rest 2	Mean RR	-16.056 (14.131)	-43.753 (-57.885, -29.621)	11.641 (-2.491, 25.772)
EPA6	BioPatch	Paced Breathing	Mean RR	-10.495 (25.385)	-60.25 (-85.637, -34.863)	39.26 (13.873, 64.647)
EPA6	BioPatch	Standing	Mean RR	-9.946 (21.481)	-52.048 (-73.531, -30.566)	32.157 (10.675, 53.639)
EPA6	BioPatch	Rest 1	SDNN	-3.446 (4.405)	-12.079 (-16.484, -7.674)	5.188 (0.783, 9.593)
EPA6	BioPatch	Stressor	SDNN	9.118 (51.193)	-91.22 (-142.415, -40.024)	109.457 (58.261, 160.652)
EPA6	BioPatch	Rest 2	SDNN	-1.466 (3.741)	-8.799 (-12.54, -5.057)	5.867 (2.126, 9.609)
EPA6	BioPatch	Paced Breathing	SDNN	-4.571 (16.113)	-36.153 (-52.267, -20.039)	27.011 (10.897, 43.125)
EPA6	BioPatch	Standing	SDNN	-7.985 (13.05)	-33.563 (-46.614, -20.512)	17.594 (4.543, 30.645)
EPA6	BioPatch	Rest 1	Mean HR	1.734 (1.869)	-1.929 (-3.798, -0.06)	5.398 (3.528, 7.267)
EPA6	BioPatch	Stressor	Mean HR	1.486 (0.698)	0.119 (-0.579, 0.817)	2.854 (2.156, 3.551)
EPA6	BioPatch	Rest 2	Mean HR	1.391 (1.189)	-0.94 (-2.129, 0.249)	3.721 (2.532, 4.91)
EPA6	BioPatch	Paced Breathing	Mean HR	0.793 (2.723)	-4.545 (-7.268, -1.821)	6.131 (3.407, 8.854)
EPA6	BioPatch	Standing	Mean HR	1.079 (2.384)	-3.594 (-5.978, -1.209)	5.752 (3.368, 8.137)
EPA6	BioPatch	Rest 1	STD HR	-0.193 (0.503)	-1.178 (-1.681, -0.676)	0.793 (0.29, 1.295)
EPA6	BioPatch	Stressor	STD HR	0.601 (3.573)	-6.401 (-9.974, -2.829)	7.603 (4.03, 11.176)
EPA6	BioPatch	Rest 2	STD HR	0.049 (0.568)	-1.064 (-1.632, -0.497)	1.162 (0.594, 1.729)
EPA6	BioPatch	Paced Breathing	STD HR	-1.163 (4.107)	-9.213 (-13.32, -5.105)	6.887 (2.78, 10.994)
EPA6	BioPatch	Standing	STD HR	-1.331 (3.339)	-7.876 (-11.215, -4.536)	5.214 (1.875, 8.554)
EPA6	BioPatch	Rest 1	RMSSD	-6.816 (7.951)	-22.399 (-30.35, -14.448)	8.768 (0.817, 16.719)
EPA6	BioPatch	Stressor	RMSSD	0.762 (34.162)	-66.196 (-100.361, -32.032)	67.721 (33.556, 101.885)
EPA6	BioPatch	Rest 2	RMSSD	-3.634 (7.244)	-17.833 (-25.077, -10.588)	10.564 (3.32, 17.809)
EPA6	BioPatch	Paced Breathing	RMSSD	-5.775 (14.717)	-34.62 (-49.337, -19.902)	23.07 (8.352, 37.787)
EPA6	BioPatch	Standing	RMSSD	-8.03 (12.319)	-32.176 (-44.496, -19.856)	16.116 (3.796, 28.436)
EPA6	BioPatch	Rest 1	LF/HF Power	1.321 (2.363)	-3.311 (-5.674, -0.948)	5.952 (3.589, 8.315)
EPA6	BioPatch	Stressor	LF/HF Power	0.671 (2.097)	-3.44 (-5.537, -1.343)	4.781 (2.684, 6.879)

Device 1	Device 2	Segment	Measure	Mean Difference (SD)	Lower Limit of Agreement (95%CI)	Upper Limit of Agreement (95% CI)
EPA6	BioPatch	Rest 2	LF/HF Power	0.714 (2.216)	-3.629 (-5.845, -1.413)	5.057 (2.841, 7.273)
EPA6	BioPatch	Paced Breathing	LF/HF Power	1.988 (5.999)	-9.77 (-15.769, -3.771)	13.745 (7.746, 19.744)
EPA6	BioPatch	Standing	LF/HF Power	4.451 (17.015)	-28.899 (-45.915, -11.882)	37.802 (20.785, 54.818)
EPA6	HeartMath Handheld	Rest 1	Mean RR	-17.631 (8.811)	-34.901 (-45.154, -24.648)	-0.36 (-10.614, 9.893)
EPA6	HeartMath Handheld	Stressor	Mean RR	-18.97 (6.409)	-31.533 (-38.991, -24.074)	-6.407 (-13.866, 1.051)
EPA6	HeartMath Handheld	Rest 2	Mean RR	-17.96 (16.432)	-50.166 (-69.286, -31.046)	14.246 (-4.874, 33.366)
EPA6	HeartMath Handheld	Paced Breathing	Mean RR	-17.092 (26.588)	-69.205 (-100.143, -38.267)	35.021 (4.083, 65.96)
EPA6	HeartMath Handheld	Standing	Mean RR	-34.346 (69.168)	-169.914 (-255.615, -84.213)	101.222 (15.521, 186.923)
EPA6	HeartMath Handheld	Rest 1	SDNN	1.079 (5.188)	-9.09 (-15.126, -3.053)	11.247 (5.21, 17.284)
EPA6	HeartMath Handheld	Stressor	SDNN	12.783 (53.783)	-92.633 (-155.216, -30.05)	118.199 (55.616, 180.781)
EPA6	HeartMath Handheld	Rest 2	SDNN	1.925 (5.534)	-8.922 (-15.361, -2.483)	12.771 (6.332, 19.21)
EPA6	HeartMath Handheld	Paced Breathing	SDNN	1.125 (8.87)	-16.26 (-26.582, -5.939)	18.51 (8.189, 28.831)
EPA6	HeartMath Handheld	Standing	SDNN	-7.487 (8.544)	-24.234 (-34.821, -13.648)	9.259 (-1.327, 19.846)
EPA6	HeartMath Handheld	Rest 1	Mean HR	1.465 (0.767)	-0.038 (-0.931, 0.854)	2.967 (2.075, 3.86)
EPA6	HeartMath Handheld	Stressor	Mean HR	1.874 (0.527)	0.841 (0.228, 1.454)	2.906 (2.293, 3.519)
EPA6	HeartMath Handheld	Rest 2	Mean HR	1.519 (1.287)	-1.004 (-2.501, 0.494)	4.042 (2.544, 5.54)
EPA6	HeartMath Handheld	Paced Breathing	Mean HR	1.534 (2.831)	-4.014 (-7.308, -0.721)	7.081 (3.788, 10.375)
EPA6	HeartMath Handheld	Standing	Mean HR	3.702 (7.545)	-11.086 (-20.434, -1.738)	18.49 (9.142, 27.838)
EPA6	HeartMath Handheld	Rest 1	STD HR	0.136 (0.437)	-0.72 (-1.228, -0.212)	0.993 (0.484, 1.501)
EPA6	HeartMath Handheld	Stressor	STD HR	0.756 (3.569)	-6.238 (-10.391, -2.086)	7.751 (3.598, 11.904)
EPA6	HeartMath Handheld	Rest 2	STD HR	0.288 (0.597)	-0.882 (-1.576, -0.187)	1.458 (0.764, 2.153)
EPA6	HeartMath Handheld	Paced Breathing	STD HR	0.185 (1.208)	-2.182 (-3.588, -0.777)	2.552 (1.146, 3.957)
EPA6	HeartMath Handheld	Standing	STD HR	-0.634 (0.687)	-1.981 (-2.832, -1.129)	0.713 (-0.139, 1.564)
EPA6	HeartMath Handheld	Rest 1	RMSSD	-0.326 (9.738)	-19.412 (-30.743, -8.081)	18.759 (7.429, 30.09)
EPA6	HeartMath Handheld	Stressor	RMSSD	-3.251 (30.798)	-63.615 (-99.451, -27.778)	57.113 (21.276, 92.949)
EPA6	HeartMath Handheld	Rest 2	RMSSD	-0.449 (7.928)	-15.987 (-25.212, -6.763)	15.089 (5.864, 24.314)
EPA6	HeartMath Handheld	Paced Breathing	RMSSD	-4.769 (15.239)	-34.637 (-52.368, -16.905)	25.098 (7.367, 42.83)
EPA6	HeartMath Handheld	Standing	RMSSD	-14.443 (12.028)	-38.018 (-52.921, -23.115)	9.132 (-5.771, 24.035)
EPA6	HeartMath Handheld	Rest 1	LF/HF Power	0.575 (1.822)	-2.995 (-5.115, -0.875)	4.146 (2.026, 6.266)
EPA6	HeartMath Handheld	Stressor	LF/HF Power	0.99 (1.524)	-1.998 (-3.771, -0.224)	3.978 (2.204, 5.751)
EPA6	HeartMath Handheld	Rest 2	LF/HF Power	0.106 (0.939)	-1.734 (-2.827, -0.641)	1.947 (0.854, 3.039)
EPA6	HeartMath Handheld	Paced Breathing	LF/HF Power	1.704 (5.086)	-8.265 (-14.183, -2.347)	11.672 (5.754, 17.59)
EPA6	HeartMath Handheld	Standing	LF/HF Power	7.392 (18.41)	-28.691 (-51.501, -5.881)	43.475 (20.665, 66.285)
EPA6	HeartMath PC	Rest 1	Mean RR	-20.648 (32.073)	-83.511 (-118.808, -48.215)	42.215 (6.919, 77.511)
EPA6	HeartMath PC	Stressor	Mean RR	-57.94 (88.052)	-230.522 (-327.423, -133.622)	114.642 (17.742, 211.543)
EPA6	HeartMath PC	Rest 2	Mean RR	-1.017 (36.408)	-72.377 (-112.444, -32.31)	70.344 (30.277, 110.411)
EPA6	HeartMath PC	Paced Breathing	Mean RR	13.655 (74.092)	-131.565 (-213.102, -50.027)	158.875 (77.337, 240.412)
EPA6	HeartMath PC	Standing	Mean RR	-40.322 (66.252)	-170.176 (-243.086, -97.266)	89.533 (16.623, 162.443)
EPA6	HeartMath PC	Rest 1	SDNN	-7.541 (16.933)	-40.729 (-59.364, -22.095)	25.647 (7.013, 44.281)

Device 1	Device 2	Segment	Measure	Mean Difference (SD)	Lower Limit of Agreement (95%CI)	Upper Limit of Agreement (95% CI)
EPA6	HeartMath PC	Stressor	SDNN	-3.552 (60.098)	-121.343 (-187.48, -55.206)	114.239 (48.103, 180.376)
EPA6	HeartMath PC	Rest 2	SDNN	-12.03 (19.223)	-49.707 (-70.862, -28.553)	25.646 (4.492, 46.801)
EPA6	HeartMath PC	Paced Breathing	SDNN	-1.955 (22.306)	-45.675 (-70.223, -21.128)	41.765 (17.217, 66.312)
EPA6	HeartMath PC	Standing	SDNN	-22.857 (20.013)	-62.083 (-84.107, -40.058)	16.369 (-5.656, 38.393)
EPA6	HeartMath PC	Rest 1	Mean HR	2.13 (2.705)	-3.172 (-6.148, -0.195)	7.432 (4.455, 10.408)
EPA6	HeartMath PC	Stressor	Mean HR	6.516 (11.552)	-16.126 (-28.838, -3.413)	29.157 (16.445, 41.87)
EPA6	HeartMath PC	Rest 2	Mean HR	0.389 (2.685)	-4.874 (-7.828, -1.919)	5.652 (2.697, 8.607)
EPA6	HeartMath PC	Paced Breathing	Mean HR	-0.632 (5.366)	-11.15 (-17.055, -5.244)	9.886 (3.981, 15.792)
EPA6	HeartMath PC	Standing	Mean HR	4.663 (7.297)	-9.639 (-17.669, -1.609)	18.964 (10.934, 26.994)
EPA6	HeartMath PC	Rest 1	STD HR	-0.548 (1.352)	-3.199 (-4.687, -1.711)	2.102 (0.614, 3.591)
EPA6	HeartMath PC	Stressor	STD HR	-0.507 (4.147)	-8.634 (-13.197, -4.071)	7.621 (3.057, 12.184)
EPA6	HeartMath PC	Rest 2	STD HR	-0.992 (1.762)	-4.445 (-6.383, -2.506)	2.461 (0.523, 4.4)
EPA6	HeartMath PC	Paced Breathing	STD HR	-0.652 (2.135)	-4.836 (-7.185, -2.487)	3.531 (1.182, 5.88)
EPA6	HeartMath PC	Standing	STD HR	-1.996 (1.743)	-5.412 (-7.33, -3.494)	1.421 (-0.498, 3.339)
EPA6	HeartMath PC	Rest 1	RMSSD	-19.378 (27.344)	-72.972 (-103.064, -42.881)	34.216 (4.124, 64.307)
EPA6	HeartMath PC	Stressor	RMSSD	-29.231 (49.569)	-126.386 (-180.937, -71.836)	67.925 (13.374, 122.475)
EPA6	HeartMath PC	Rest 2	RMSSD	-30.065 (26.866)	-82.723 (-112.29, -53.157)	22.593 (-6.973, 52.16)
EPA6	HeartMath PC	Paced Breathing	RMSSD	-29.77 (29.396)	-87.385 (-119.735, -55.036)	27.845 (-4.504, 60.195)
EPA6	HeartMath PC	Standing	RMSSD	-48.421 (30.359)	-107.924 (-141.334, -74.514)	11.082 (-22.327, 44.492)
EPA6	HeartMath PC	Rest 1	LF/HF Power	1.877 (2.753)	-3.518 (-6.548, -0.489)	7.272 (4.243, 10.301)
EPA6	HeartMath PC	Stressor	LF/HF Power	2.768 (3.203)	-3.511 (-7.037, 0.014)	9.046 (5.521, 12.572)
EPA6	HeartMath PC	Rest 2	LF/HF Power	1.484 (1.633)	-1.717 (-3.514, 0.08)	4.685 (2.888, 6.483)
EPA6	HeartMath PC	Paced Breathing	LF/HF Power	6.028 (5.363)	-4.483 (-10.385, 1.419)	16.54 (10.638, 22.441)
EPA6	HeartMath PC	Standing	LF/HF Power	8.278 (12.387)	-16.001 (-29.633, -2.369)	32.557 (18.925, 46.189)
